# Treading a HOSTile path: Mapping the dynamic landscape of host cell–rotavirus interactions to explore novel host-directed curative dimensions

**DOI:** 10.1080/21505594.2021.1903198

**Published:** 2021-04-05

**Authors:** Upayan Patra, Urbi Mukhopadhyay, Arpita Mukherjee, Shanta Dutta, Mamta Chawla-Sarkar

**Affiliations:** a Division of Virology, National Institute of Cholera and Enteric Diseases, Beliaghata, Kolkata, India; b Division of Bacteriology, National Institute of Cholera and Enteric Diseases, Beliaghata, Kolkata, India

**Keywords:** Rotavirus, host–virus interactions, pro-viral and antiviral host determinants, host-directed antivirals

## Abstract

Viruses are intracellular pathogens and are dependent on host cellular resources to carry out their cycles of perpetuation. Obtaining an integrative view of host–virus interaction is of utmost importance to understand the complex and dynamic interplay between viral components and host machineries. Besides its obvious scholarly significance, a comprehensive host–virus interaction profile also provides a platform where from host determinants of pro-viral and antiviral importance can be identified and further be subjected to therapeutic intervention. Therefore, adjunct to conventional methods of prophylactic vaccination and virus-directed antivirals, this host-targeted antiviral approach holds promising therapeutic potential. In this review, we present a comprehensive landscape of host cellular reprogramming in response to infection with rotavirus (RV) which causes profuse watery diarrhea in neonates and infants. In addition, an emphasis is given on how host determinants are either usurped or subverted by RV in course of infection and how therapeutic manipulation of specific host factors can effectively modulate the RV life cycle.

## Introduction

Rotaviruses (RVs), belonging to the family *Reoviridae*, are the causative agents of acute watery diarrhea. Children below 5 years of age and young animals are particularly vulnerable to infection with RVs through fecal-oral transmission chain; however, immunosuppressed human adults may also acquire infection. Clinical symptoms of RV infection, which appear after a short incubation period of 2–3 days, include vomiting, nausea, and profuse watery diarrhea. Severe dehydration due to rotaviral diarrhea requires prompt medical intervention mainly in the form of fluid and electrolyte replenishment. Rotaviral gastroenteritis accounts for a staggering death toll and associated morbidities especially in countries with compromised socio-economic conditions [1–5]. Introduction of anti-rotaviral vaccination has successfully curtailed disease prevalence in developed countries, but yielded sub-optimal efficacy in developing nations of endemic settings with high viral disease burden and inter-specific re-assortment rates [6]. With ever-increasing viral heterogeneity, prophylactic vaccination as the only mode of disease prevention might be fallible in the long run because of potential emergence of escape-mutants. Targeting mutation-prone viral proteins with antivirals may further aggravate the scenario by inadvertently selecting drug-resistant viral strains. Thus, adjunct to vaccines, there is a demanding need for developing anti-rotaviral drugs targeted against non-mutable host determinants of infection. Like any other virus, RVs induce extensive reprogramming of host cellular homeostasis upon infection. Core to virus-induced host cell take over process have been evasion of innate antiviral measures (constituted by antiviral host cellular determinants) and exploitation of cellular machineries (pro-viral host cellular determinants), often non-canonically, to convert an apparently hostile host environment into a favorable one conducive to viral perpetuation. Because of obligations of viruses to usurp pro-viral host determinants at the expense of antagonizing antiviral host determinants for successful completion of replication cycles, cellular factors have emerged as sensitive drug targets for treating viral infection. Therefore, small molecules with antagonistic activity against pro-viral host determinants and/or agonistic activity toward antiviral host determinants can potentially interfere with the viral life cycle events leading to impairment of viral infection. Not surprisingly, dissecting host-RV interactions has yielded a number of crucial host components, experimental manipulations of which have been reported to heavily influence RV infectivity. In this review, different modalities of host-RV interactions are described and potential avenues for host-targeted intervention strategies of anti-RV importance are discussed. Many of such host machineries regulate viral life cycle events in mutually interdependent ways. Moreover, with the promising potentials of drug repurposing, host-directed intervention approach may provide an important dimension to designing anti-rotaviral therapeutics of significant clinical importance. Druggable anti-rotaviral host candidates can also be explored for their antiviral relevance in general for other viruses.

## Life cycle of rotavirus

The infectious unit of RV is a non-enveloped triple-layered particle (TLP) with three concentric proteinaceous capsid layers. The innermost core shell is formed of the RV structural protein VP2 and encapsidates the 11 segments of rotaviral double-stranded RNA (dsRNA) genome along with two other structural proteins VP1 (the viral RNA dependent RNA polymerase) and VP3 (the viral mRNA capping enzyme) [7–9]. The middle layer consists of trimers of the structural protein VP6 and serves as a tether between the innermost and the outermost layer. Based on the genetic variability of VP6, 10 species of RVs have been identified to date (RV A-J) [10–12], with RV A being the most common type infecting humans. The outermost layer consisting of spike proteins VP4 embedded in glycoproteinaceous shell of VP7 is involved in the viral attachment to and penetration within the host cells (primarily enterocytes). Group A RVs have been classified on the basis of genetic architecture of VP7 (G types; G stands for Glycosylated) and VP4 (P types; P stands for Protease-sensitive) genes. Based on the G-P typing, 36 G and 51 P types have been identified yet in human and animal species, globally [13].

**Figure 1.**
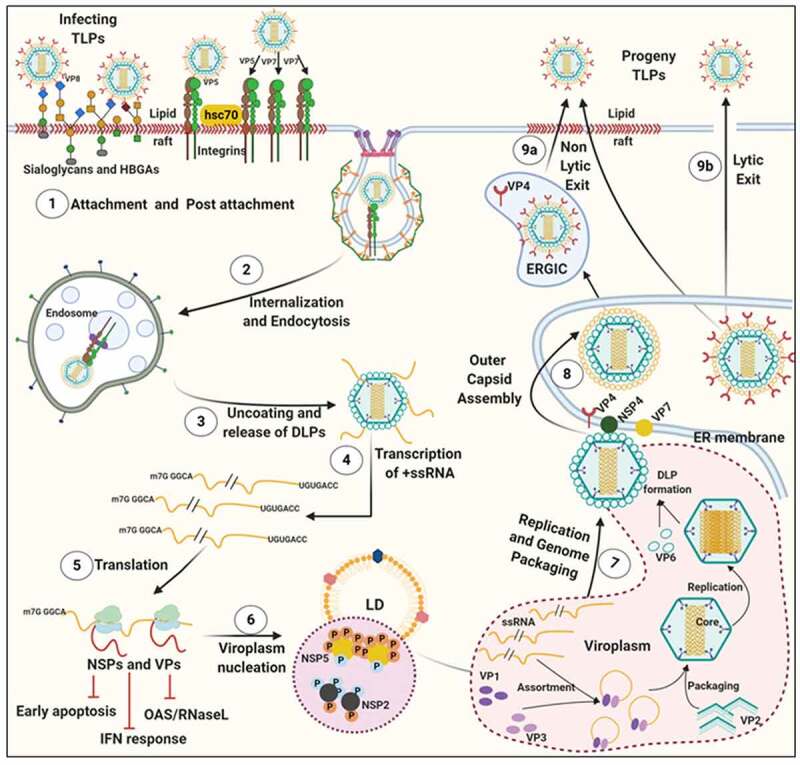
**Life cycle of RV**. (1) The invading RV TLPs attach to receptors [sialoglycans and Histo-blood group antigens (HBGAs)] and co-receptors [integrins and heat shock cognate protein 70 (hsc70)] assembled in lipid rafts on host cellular plasma membrane. (2) RV TLPs are endocytosed and trafficked into the cells. (3) TLPs are uncoated to form DLPs which are released from endosomes into the host cellular cytoplasm. (4) Within DLPs, viral RNAs are transcribed to yield capped, (+)ssRNAs. (5) RV (+)ssRNAs are translated on host cellular ribosomes to synthesize viral proteins (NSPs and VPs) which are necessary for evading innate antiviral immune response and (6) nucleation of RV-specific inclusion bodies called viroplasms. Host cellular lipid droplets (LDs) act as scaffolds for viroplasm formation. (7) Inside the maturing viroplasms, viral genome replication takes place within the VP2-encaged viral cores through the VP2-driven polymerase activity of VP1. Progeny cores acquire the VP6 layer and form progeny DLPs which may either amplify the replication cycle by producing secondary transcripts or (8) enter into the morphogenetic assembly pathway. Acquisition of the outer capsid occurs by a budding step through the ER-derived cellular membrane where VP6 on DLPs docks on NSP4 on ER-derived membrane. Subsequently, NSP4 is stripped and VP7-VP4 layer is assembled. Alternatively, acquisition of VP4 spikes may occur on VP7-surrounded virions within ER-Golgi intermediate compartment (ERGIC)/plasma membrane lipid raft domains (9a) before non-lytic virion release. (9b) RV progenies may also exit through lytic mechanisms

RV TLPs primarily infect intestinal epithelial cells and possess a lytic replication cycle that initiates with viral entry which includes attachment and post-attachment interactions on the host cell membrane, followed by internalization, endosomal trafficking, and subsequent penetration of the virus into the host cell cytoplasm in the form of partially unmasked double-layered particles (DLPs) (Figure 1). To gain infectivity, VP4 undergoes a specific proteolytic cleavage by trypsin to form 2 cleavage products VP8 and VP5, both of which along with the VP7, are essential for initial attachment and post-attachment interactions with the host cells [14]. Once inside the cytoplasm, VP1, with the assistance of VP2 and VP3, initiates transcription within DLPs to form capped, non-polyadenylated, positive-sense single-stranded RNAs [(+)ssRNAs] which subsequently act as messengers for viral protein translation (six structural proteins VP1-4, VP6, VP7 and six non-structural proteins NSP1-6) (Figure 1) [15–17]. Initial synthesis of viral proteins is necessary for the subversion of host innate immune response which primarily entails antagonizing antiviral Interferon (IFN) response and host cellular apoptosis by NSP1 as well as subversion of the 2ʹ, 5ʹ-oligoadenylate synthetase (OAS)/RNase L pathway by VP3 (Figure 1) [18]. Primary synthesis of two other non-structural proteins, NSP2 and NSP5, plays a crucial role in nucleation of membrane-free, polyribosome-surrounded dynamic inclusion bodies called viroplasms which subsequently accumulate other RV proteins VP1, VP2, VP3, VP6 as well as (+)ssRNAs and dsRNAs (Figure 1) [19–21]. These viral inclusion bodies serve as the safe-houses for rotaviral genome replication. Replication of the viral genome includes mutual affinity-driven formation of the VP1–VP3–(+)ssRNA complexes within the decameric VP2 assembly core and subsequent initiation of the (−) strand RNA synthesis through the VP2-driven polymerase activity of VP1 (Figure 1) [22–24]. The resulting 11 dsRNA genome segments within the progeny cores acquire a peripheral layer of VP6 to form progeny DLPs which can further amplify the replication cycle by producing secondary transcripts or may enter into the morphogenetic assembly pathway [25,26]. Acquirement of the outer capsid layer by the immature DLPs residing within viroplasms to form mature infectious TLPs is the prime most important step in RV morphogenesis. Unlike most of the non-enveloped viruses, RV requires a budding step through ER-derived cellular membranes where VP6 on DLPs docks on NSP4 on ER-derived membranes (Figure 1) [27–29] along with co-recruitment of VP4 and VP7 on NSP4 [30–33]. Besides taking part in morphogenesis, NSP4 has been shown to be the exclusive viral component for RV-induced diarrheal pathophysiology [34]. The final part of the morphogenesis includes budding of the DLP–VP7-VP4–NSP4 complex into the lumen of the ER-derived membranes, stripping of the NSP4-containing ER envelope, and assembly of the VP7 outer layer, thereby locking VP4 into correct places (Figure 1) [29,35]. An assembly model with VP4 organization as a post-ER event has also been put forward (Figure 1) [36,37]. Progeny TLPs exit infected cells either through lytic mechanisms or by non-lytic secretory pathways which bypass the involvement of Golgi apparatus and lysosomes [38,39] to continue successive waves of infection (Figure 1).

## Involvement of host machineries during rotaviral life cycle

Interestingly, apart from viral contributors, a plethora of host components are integral to the dynamic interfaces between host cells and RV, which finally shape the outcome of infection and pathophysiology. In the following sections, we will describe different modalities of host-RV interactions from three principal aspects: i) exploiting host determinates to enable entry of virions, ii) evasion of antiviral innate immune response to establish a pro-viral host cellular atmosphere, and lastly iii) usurpation of host machineries to favor viral life cycle events such as translation, transcription, replication, viroplasm formation, and morphogenesis. In addition to providing a comprehensive network of host–RV interactions, we will also highlight probable modes of therapeutic interventions by targeting host determinants of infection.

## Exploiting host determinates to enable entry of virions

### Sialylated and fucosylated glycans: Tethering virions to host cell membrane receptors

**Figure 2.**
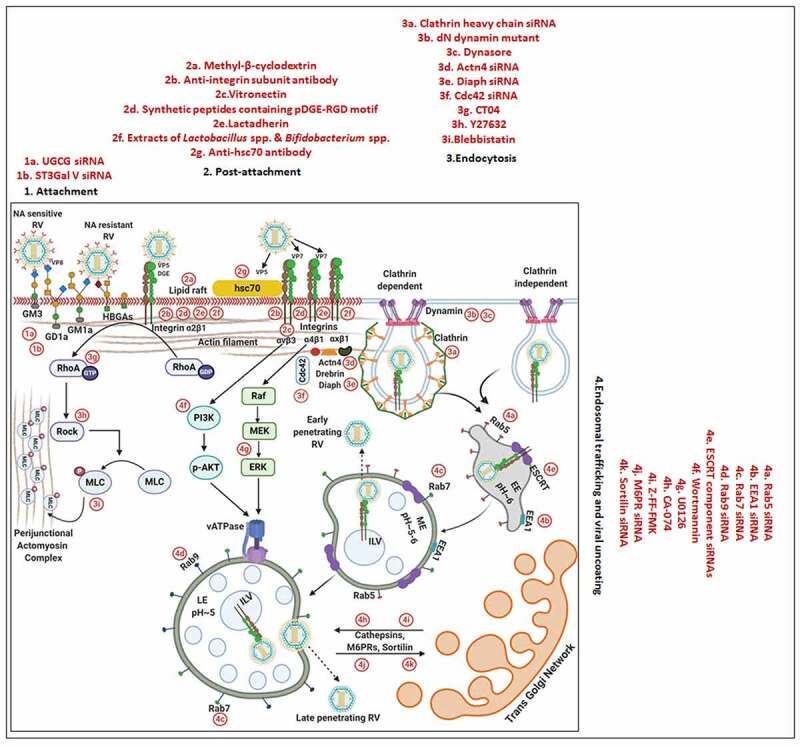
**Entry of RV TLPs into host cells**. The entire event of ingress of RV TLPs across the host cell membrane into the cytoplasm is an elaborate process which consists of (1) attachment and (2) post-attachment interactions between the virions and the host cells, followed by (3) endocytosis, (4) endosomal trafficking and penetration of the virus into the host cytosol in the form of DLPs. (1) Host cell membrane sialylated (SA-containing) and fucosylated (HBGAs) glycans act as receptors for RV TLPs. NA-sensitive RV strains require terminal SA-containing glycans as entry receptors; NA-resistant RV strains utilize sub-terminal SA-containing glycans and HBGAs. The RV spike protein VP4, especially by its trypsin-digested domain VP8, interacts with the cellular receptors. (2) Cellular integrins (α_2_β_1_, α_4_β_1_, α_X_β_2_, α_V_β_3_) and hsc70 act as post-attachment co-receptors for RV TLPs. A DGE motif of RV-VP5 which is a trypsinized fragment of RV-VP4 is required for interaction with α_2_β_1_ integrin. Additional VP5 and VP7 regions are required for interactions with hsc70 and integrins. Cellular receptors and co-receptors for RV entry are spatially assembled in lipid raft microdomains of cell membrane. (3) Virions are subsequently internalized by clathrin-mediated or clathrin-independent endocytosis. The GTPase dynamin, cellular actin microfilaments, several actin-binding proteins (Actn4, Diaph, drebrin), and actin microfilament regulatory components (RhoA, Cdc42) modulate the process of rotaviral entry. Viral tethering activates RhoA-dependent stress fiber formation and TJ disruption downstream of RhoA/ROCK/MLC signaling. (4) Endocytosed virions converge in EEs and may progress on to the LEs for uncoating and penetration (late penetrating RVs) or may be released before that (early penetrating RVs). Therefore, early penetrating RVs are sensitive to EE markers EEA1 and Rab5 but not to LE markers Rab7 and Rab9 whereas late penetrating RVs lose infectivity in absence of Rab7 and Rab9. ESCRT complex responsible for ILV formation is also essential for RV entry process. Late penetrating RV strains require protein transporters CD-M6PRs which deliver cathepsins from TGN to LEs. Other protein transporters such as CI-M6PRs and sortilin also regulate infectivity of late penetrating RV strains. TLPs are uncoated to form DLPs which are released from endosomal compartment into host cytosol. Endosomal acidification and calcium concentration can act as triggers for viral nucleocapsid exit. PI3K and ERK signaling downstream of interaction between cell surface receptor/co-receptors and late penetrating RV strains can activate the V-ATPase proton pump resulting in endosomal acidification and viral uncoating. Several inhibitors acting on different steps of RV entry are shown. For additional details, please refer the text

Based on the sensitivity of RV infectivity to neuraminidase (NA) pre-treatment of host cells, RV strains were initially categorized into NA-sensitive [which were postulated to require sialic acid (SA) residues of host cell gangliosides for attachment as in case of some animal RV strains] or NA-resistant (such as a few animal and most human RV strains which were thought to be independent of host cell SA moieties for initial tethering) groups [14]. The NA-resistant RV strains were subsequently found out to be those which either bind to NA-insensitive internal (sub-terminal) SA residues on host cell surface or dock on human histo-blood group antigens (HBGAs); NA-sensitivity of some RV strains is also justified as it requires host cell gangliosides with terminally exposed SA residues [40–42]. Interestingly, knocking down expression of UDP-glucose:ceramide glucosyltransferase (UGCG) and lactosyl ceramide-α-2,3-sialyl transferase 5 (ST3Gal V), two key enzymes belonging to the ganglioside synthesis pathway, in MA104 cell line by RNA interference (RNAi) effectively decreased cellular ganglioside levels and reduced infectivity of all RV strains (NA-resistant as well as NA-sensitive) examined [43], clearly indicating importance of host cell surface gangliosides for RV infection (Figure 2).

The VP8 domain of VP4 has been implicated to tether the viral particle to the cell surface (Figure 2). The tethering requires interaction of the SA-binding domain of VP8 with the SA residues of gangliosides on host cell surface [44,45]. The crystal complex of VP8 from NA-sensitive RV strains with SA revealed SA binding near the cleft region of VP8. The cleft of VP8 from NA-resistant RV strains has been proposed to be wider to allow binding of gangliosides with sub-terminal SA residues [42,46].

Fucosylated human histo-blood group antigens (HBGAs), such as H-type glycans and A-type glycans, have also been implicated for host cell attachment in case of many human RV strains (Figure 2) [47–50]. The crystal structure from a [P14] VP8 revealed that it has a narrow cleft (as observed in NA-sensitive RV strains), and that the A-type HBGA docks on the same region of the cleft where SA docks on the animal VP8 [46]. Notably, the occurrence of age-restricted infectivity by neonatal RV strains can partially be attributed to varying glycan modification observed during neonatal development. There also exists a correlation between the VP4 genotype of the infecting RV strain and the HBGA phenotype as well as the secretory status of the infected neonates as secretors [with wild type Galactoside alpha-(1,2)-fucosyltransferase 2 (FUT2) enzyme] have been shown to be vulnerable to RV infection (at least for VP4 genotype P [8]) whereas non-secretors (with genetically mutated FUT2) innately protected [51–53].

Interestingly, human milk oligosaccharides (HMOs), especially their sialylated and fucosylated structural variants have been reported to exert anti-RV potential in a VP4 genotype–specific way [54,55]. Mechanisms of such antiviral activities have been poorly defined but are hypothesized to be virion-targeted where these HMOs may act as soluble decoy receptors to competitively inhibit attachment of RVs to host cellular glycan receptors; secondary implications of HMO-mediated regulation on host cellular apoptosis have also been put forward [54,56]. In light of a recent finding, however, there has been a paradigm shift in the current understanding of the impacts of HMO on RV infectivity where an additional interplay between milk microbiome and infant gut microbiome may shape the outcome of neonatal RV infection [57]. In this report, HMOs have been shown to foster infection of a particular neonatal RV strain G10P [11] and also of a currently licensed vaccine strain (G9P [11], strain 116E; Rotavac®), warranting careful reevaluation for inclusion of specific HMOs in infant formulae [57].

### Integrins and heat shock cognate protein 70: Regulating post-attachment interactions

Initial tethering of infectious virions to their cognate receptors on host cell surface is followed by specific post-attachment interactions between the host and the virions before the virions gain competence for cellular entry. The prime most cellular contributors for enabling efficient post-attachment interactions are several integrins (α_2_β_1_, α_4_β_1_, α_X_β_2_, α_V_β_3_) and the heat shock cognate protein 70 (hsc70), which along with the gangliosides (required for initial attachment) and the infectious viral particles often spatially assemble in the detergent-resistant membrane domains called lipid rafts during infection (Figure 2) [58]. Not surprisingly, therefore, lipid raft destabilization (through membrane cholesterol depletion by Methyl-β-cyclodextrin) has been associated with reduced infectivity of many RV strains at the post-attachment entry stage, clearly emphasizing the significance of lipid raft integrity for RV infection (Figure 2) [59–62].

Detailed investigations further revealed RV-integrin interaction to involve a DGE motif near the amino-terminal end of the VP5 domain of VP4 and the domain I of the integrin subunit α_2_ within α_2_β_1_ (Figure 2) [63,64]. In case of interaction with integrin α_V_β_3_, however, a linear sequence in RV VP7 has been implicated [63,64]. Indeed, incubation of cells prior to infection with blocking antibodies against integrin subunits (such as α_2_, α_4_, α_v_, β_2_, β_3_), integrin ligands (such as vitronectin for α_v_β_3_), synthetic peptides (pDGE-RGD containing viral sequence motif DGE and canonical integrin-binding motif RGD), lactadherin (with DGE motif), and even probiotic extracts of *Lactobacillus* spp. and *Bifidobacterium* spp. with integrin (β_3_) binding capability blocked RV infectivity at the post-attachment step (Figure 2) [61,65,66]. Moreover, the strain-specific anti-RV effects of the flavonoid genistein (but not of its inactive analogue daidzein) at the virus-host cell surface attachment stage has been explained to possibly stem from inhibition of integrin activation though the protein tyrosine kinase inhibition activity of genistein [67]. Of note, transcriptional regulation of integrins (both RV co-receptors and non-receptor integrins) was reported as a result of RV replication-mediated Phosphoinositide 3-Kinase (PI3K) activation but independent of RV-integrin interaction [68]. Interestingly, integrins are generally localized on the basolateral cell membrane whereas RVs primarily infect mature enterocytes at the tip of the small intestinal villi. This apparent paradox can be explained from the observation that a recombinant VP8 protein of RRV was shown to loosen the integrity of intercellular tight junctions leading to a reduction in the transepithelial electrical resistance of polarized Madin–Darby canine kidney (MDCK) cells and allowing re-positioning of many basolateral proteins (such as integrins α_V_β_3_, β_1_, and the Na^+^-K^+^-ATPase) on the apical side of the cells [69]. Recent reports have also advocated for the direct involvement of the tight-junction proteins Junction Adhesion Molecule A (JAM-A), occludin, and Zonula occludens protein 1 (ZO-1) for entry of some RV strains, where JAM-A was specifically shown to function as a co-receptor for RV VP4 [70].

Unlike integrins which are dispensable for some RV strains, hsc70 has been shown to be required for all strains of RV tested for establishing efficient infection [62,71]. A region spanning from amino acids 642 to 659 on RV VP5 proved essential for interaction with hsc70 [72]; a synthetic peptide which corresponds to this region as well as hsc70-specific monoclonal antibody blocked virus infectivity but not viral attachment to host cells, suggesting requirement of hsc70 at a post-attachment step (Figure 2) [71,72]. Probiotic extracts of *Lactobacillus* spp. and *Bifidobacterium* spp. also competitively inhibited virion attachment to hsc70 (Figure 2) [66]. The implication of probiotics to regulate rotaviral infectivity is of particular interest as evidence is emerging in favor of transkingdom interactions involving intestinal bacterial microbiota in a complex web with helminths, phages, and fungal population to shape antiviral immunity *in vivo* [73]. In another study, the ATPase domain of hsc70 was shown to facilitate conformational changes in the virions in favor of viral entry [74]. Notably, the importance of lipid raft-associated oxidoreductase protein disulfide isomerase (PDI) was also demonstrated to facilitate RV entry as inhibition of PDI redox activity (by cell membrane-impermeant thiol/disulfide-reactive agents such as DTNB [5,5-dithio-bis-(2-nitrobenzoic acid)] and bacitracin) reduced RV infectivity [75].

Of importance, targeted inhibition of specific host receptors and co-receptors (achieved experimentally by different approaches such as protease treatment, antibody/peptide/sugar analogue-mediated neutralization, RNAi) was only efficient to reduce viral infectivity by less than a log, suggesting redundancy of cellular factors utilized by RV for cellular entry and even implying the presence of yet-to-be identified host factors. Moreover, though certain sequentiality has been reported for RRV [14,76], whether the events of attachment and post-attachment interactions are sequential, alternative, or concerted have remained elusive for most RV strains.

### Clathrin, dynamin, and cytoskeletal microfilaments: Facilitating virion engulfment

Though a direct penetration of RV virions at the plasma membrane was proposed initially, with the advent of host-targeted studies using pharmacological inhibitors against endocytosis, overexpressing dominant-negative mutants, and knocking down expression of specific endocytic proteins by RNAi, importance of endocytosis in mediating virion internalization has been established (Figure 2) [62]. Based on the sensitivity of viral entry to inhibition of clathrin-mediated endocytosis (by hypertonic sucrose medium which causes dissociation of clathrin vesicles from the plasma membrane or by targeting clathrin heavy chain through RNAi), the choice of endocytic pathway proved to be RV strain-specific: clathrin-mediated endocytosis for human RV strains DS-1, Wa, WI69, and animal RV strains YM, UK, SA11-4S, nar3 (a RRV mutant) whereas clathrin- and caveolin-independent endocytic mechanism in case of RRV (Figure 2) [62,77–79]. Mortalin, belonging to the mitochondrial protein import machinery, proved to be a negative regulator of clathrin, and mortalin-overexpressed cells reduced RV infectivity at the viral entry stage [80]. It is noteworthy that NA-resistant, NA-sensitive, as well as the HBGAs-interacting RV strains can use clathrin-dependent internalization mechanism [77]. Interestingly, an importance of RV VP4 for the selection of endocytic pathway has been put forward where the substitution of a single amino acid (K187R) in the VP8 region of RRV (a mutant RRV called nar3) proved enough to shift the choice of internalization from clathrin-independent in RRV to clathrin-dependent in nar3 [77,81]. Moreover, the requirement for cholesterol and dynamin, a GTPase associated with membrane scission events and endocytosis, was revealed to be important for clathrin-dependent endocytosis of RVs into MA104 cells as both depletion of membrane cholesterol pool (by Methyl-β-cyclodextrin) and dynamin inhibition (through overexpression of dominant negative dynamin mutant) curtailed infectivity at the entry stage of all the RV strains tested (Figure 2) [62]. There are, however, contrasting reports of dynamin-dependency for RRV [62,78,79,82,83], which can be attributable to differences in the cell lines used and the methods undertaken for asserting the role of dynamin on RV infectivity.

Besides clathrins and dynamins, host microfilaments, especially the actin cytoskeletal network, have also been demonstrated to have pro-rotaviral implications during the viral entry stage. Changes in microfilaments in the form of stress fiber formation were evidenced at the very early stage of infection and were possibly triggered because of viral tethering to the host cell surface integrins and subsequent Ras homolog family member A (RhoA) activation [84]. Reorganization of actin cytoskeleton during later phase of RV infection, however, was found to be Ca^2+^-dependent and therefore sensitive to Ca^2+^ chelation (by BAPTA-AM) or NSP4 silencing [84]. Interestingly, a recent report highlighted a strong correlation between the disruption of tight junction (TJ) integrity (as evidenced by decreased transepithelial resistance and increased paracellular permeability) and the activation of the RhoA/Rho-associated protein kinase (ROCK)/Myosin light chain (MLC) signaling pathway in polarized MDCK cells during early hours of RV infection [85]. Detailed mechanistic investigations revealed virion tethering to cognate host cell surface receptors to initiate the RhoA/ROCK/MLC cascade which further leads to TJ protein (JAM-A, occluding, ZO-1) re-distribution and TJ disruption via contraction of the perijunctional actomyosin ring (Figure 2). Severance of TJ integrity is how RV virions are postulated to gain access to their post-attachment receptors such as JAM-A, occludin and ZO-1. Indeed, inhibition of the RhoA/ROCK/MLC signaling pathway using targeted small molecules (RhoA inhibitor CT04, ROCK inhibitor Y27632, MLC inhibitor blebbistatin) restored TJ integrity and prevented RV-induced TJ permeability in polarized epithelial cells, ultimately resulting in reduced production of progeny viruses (Figure 2) [85].

Apart from direct involvement of actin microfilaments, several actin-binding proteins have been reported too to influence RV infectivity at the viral entry stage (Figure 2). One such example is a VP4 interacting protein drebrin which was found to interact with cortactin at the actin filaments leading to suppression of dynamin-dependent RV endocytosis. Concomitantly, blocking drebrin function by RNAi, Clusters of regularly interspaced short palindromic repeats (CRISPR) knockout, or by chemical inhibition (by BTP-2) markedly increased host cell susceptibility to RV infection [86]. Moreover, enhanced RV infectivity associated with loss-of-function of drebrin was found to be significantly reduced when cortactin (through RNAi) and/or dynamin (by a small molecule dynasore), specifically dynamin-2 (by Dyngo-4a), were co-inhibited prior to infection [86]. RNAi-mediated loss-of-function studies have also recently vouched for considerable implications of other endocytic regulators such as actin-binding proteins Actinin 4, Diaph, and the small GTPase Cell division Control protein 42 homolog (Cdc42), as well as the Cdc42 activator Cdc42 GTPase-activating protein (CdGAP), in the process of host cell entry by RVs (specifically during the post-attachment internalization process) (Figure 2) [79,81,87].

### The endosomal network: Implications in intracellular trafficking of virions

Internalized endocytic vesicles containing the viral cargos are trafficked through the host endosomal network before double-layered viral particles are released into the cell cytoplasm. The endosomal network consists of distinct membranous compartments such as early endosomes (EEs), maturing endosomes (MEs), late endosomes (LEs), recycling endosomes (REs), and lysosomes, each of which has signature structure and localization, protein/lipid composition, luminal pH, and distinctive surface Rab GTPase. Rab proteins belong to a large family of small GTPases which regulate intracellular vesicle trafficking via recruitment of effector proteins [88,89]. Irrespective of attachment/post-attachment interactions and mode of internalization, all RV strains examined converge in EEs during the entry process as their infectivity relies on the presence of EE markers Rab5 and early endosomal antigen 1 (EEA1) (Figure 2) [79,81,90]. During fusion with the EEs, the endocytic cargos harboring the RV particles interact with the components of endosomal sorting complex required for transport (ESCRT) machinery (specifically HRS, TSG101, VPS25, VPS24, and VPS32). The ESCRT machinery, consisting primarily of four complexes-ESCRT-0, -I, -II and -III, as well as several accessory constituents, are essential for the characteristic formation of intraluminal vesicles (ILVs) within endosomes and regulate a variety of physiological and pathological processes such as endocytosis of specific cargos, receptor downregulation and retroviral budding [91,92]. Interestingly, dependency of RVs on functional ESCRT machinery in MA104 and Caco-2 cells was revealed when RNAi-based targeted silencing of ESCRT complex components reduced RV infectivity (Figure 2) [79]. Moreover, inhibition of ILV formation either by silencing VPS4A, the ESCRT-associated ATPase involved in membrane fission, or by antibody-mediated blocking of phospholipid lysobisphosphatidic acid diminished RV infectivity, suggesting importance of ILVs during rotaviral entry [79]. Requirement of VPS4A can be explained as it may help the viral particle to get internalized into the endosomal lumen. RV strains RRV and SA11, regarded as early-penetrating RVs, are released as DLPs from EEs and therefore insensitive to depletion of Rab7 which is localized to both MEs and LEs. On the other hand, rest of the RV strains which are considered as late-penetrating traffic through the endosomal network to reach LEs and therefore were revealed to be Rab7-dependent (Figure 2) [79,81]. Interestingly, a role of RV VP4 has been implicated in the differential exit of RRV and UK from endosomal network [77].

Apart from Rab7-dependency, late-penetrating RV strains such as UK, Wa, WI61, DS-1, and YM also revealed dependency on Rab9, a LE marker, and protein transporters such as cation-dependent mannose-6-phosphate receptors (CD-M6PRs), cation-independent M6PRs (CI-M6PRs), and sortilin-1 for infectivity, specifically at the viral entry stage (Figure 2) [81,93]; for RRV mutant nar3, however, CD-M6PRs, but not Rab9, proved dispensable [81]. These protein transporters deliver lysosomal acid hydrolases (such as cysteine cathepsins) as well as other non-enzymatic proteins from trans-Golgi network (TGN) to LEs and are further recycled back to TGN with the help of small GTPases Rab9 on LEs [94,95]. Interestingly, a decrease in the infectivity of late penetrating RV strains (UK, Wa, WI61, DS-1, and YM), but not of RV RRV or nar3, has been evidenced in cells where cathepsin B and L were inhibited prior to infection pharmacologically (CA-074 targeting cathepsin B, Z-FF-FMK targeting cathepsin L, leupeptin targeting pan-endolysosomal proteases) or by RNAi [81,93] (Figure 2), suggesting late penetrating RVs to require cathepsin activity for cellular entry. The exact mode of cathepsin action, however, has remained elusive.

### An interplay between endosomal acidification and conformational change of virion outer capsid: Enabling viral penetration and uncoating

Releasing from endosomal network involves uncoating of the TLP to yield DLP into the host cell cytoplasm. The exact mechanism by which DLPs are expelled from the endosomal compartments has not been identified yet. Several possible triggers such as changes in luminal pH, membrane components, calcium concentration, and lysosomal hydrolases, either acting alone or in combination, have been implicated to induce specific conformational changes in the intra-endosomal virion in favor of the viral nucleocapsid exit into cytoplasm (Figure 2) [76,96]. Interpreting cryo-electron microscopy and crystallography-based data of the VP5 domain of VP4 [97] has put forward a theory where a conformational change in the intra-endosomal RV VP4, driven by an unknown factor, results in calcium dissipation from the endosomal compartment to the cytosol which further leads to the disassembly of the VP7 smooth surface layer. This calcium leakage coupled to a lowering of intra-endosomal pH in turn triggers a conformational rearrangement of VP5 to a “fold-back” orientation thereby exposing a hydrophobic domain on VP5. Subsequent interaction of VP5 with the endosomal membrane enables disruption of the membrane leading to escape of the double-layered virus particles into the cytosol [9]. Indeed, for the early-penetrating RV strain RRV which exits endosomal network from EEs, co-localization of Rab5, an EE marker, with the “fold-back” conformation of VP5 was evidenced by confocal microscopy [83]. This observation supports the hypothesis that specific conformational changes in VP5 (and possibly VP7), triggered by the residing endosomal environment (EEs for the early-penetrating RVs; LEs for the late-penetrating RVs), facilitate penetration of the viral particles into the cytoplasm. Chemical intervention of endosomal acidification (with ammonium chloride and Bafilomycin A1) prior to infection significantly curtailed infectivity of late penetrating RV strains TFR-41, Wa, and Uk [62]; for entry of the early penetrating strain RRV, however, only sensitivity to Bafilomycin A1 (because of Bafilomycin A1-induced secondary effects rather than pH change itself), but not to ammonium chloride, has been reported [62,83]. A recent study also substantiated this finding where activated cellular kinases PI3K, Akt, and Extracellular-signal-Regulated Kinase (ERK), downstream of interaction between cell surface receptor/co-receptors and late penetrating RV strains (DS-1 and NCDV), interacted with and activated the subunit E of vacuolar-H^+^ ATPase (V-ATPase) proton pump resulting in endosomal acidification and viral uncoating [98]. Concomitantly, targeted inhibition of these cellular kinases prior to infection (PI3K/Akt pathway by wortmannin and MEK/ERK pathway by U0126) blocked release of DS-1 and NCDV from late endosomal compartment by perturbing endosomal acidification process (Figure 2) [98]. Moreover, 25-hydroxycholesterol (25HC) and 27-hydroxycholesterol (27HC), which are produced physiologically through enzymatic oxidation of cholesterol, have been shown to curtail RV infectivity in a strain independent manner by specifically inhibiting the step of viral penetration and uncoating from late endosomal compartment; mechanistically, presence of oxysterols were shown to perturb the interaction between oxysterol binding protein (OSBP) and the vesicle-associated membrane protein-associated protein A (VAP-A) leading to pronounced accumulation of cholesterol within these vesicular compartments. A small molecule U18666A (an amphipathic steroid 3-β-[2-(diethylamine)ethoxy] androst-5-en-17-one) which blocks free cholesterol exit from LEs also mimicked anti-RV effects of 25HC and 27HC at the penetration step of the RV life cycle [99]. Though the exact significance of intra-endosomal cholesterol accumulation on rotaviral uncoating process has not been addressed directly, cholesterol-rich intra-endosomal niches were reported to jeopardize protein sorting and trafficking (such as trafficking of CD-M6PRs) [100], which are proven pro-viral determinants of RV infectivity [81].

## Evasion of antiviral innate immune response

### Host pattern recognition receptors and IFN-signaling cascade

Activation of IFN-mediated primary antiviral defense includes two steps: an initial step of secretory IFN induction downstream of sensing viral pathogen-associated molecular patterns (PAMPs) by host pattern recognition receptors (PRRs) and mobilization of a conserved signaling cascade, and a second signal amplification step through autocrine and paracrine actions where secreted IFNs bind to their cognate receptors to evoke Janus kinase (JAK)- Signal transducer and activator of transcription (STAT) signal transduction leading to STAT-mediated transcriptional augmentation of antiviral ISGs [101].

Countering potential deleterious effects of host antiviral IFN-signaling and amplification has been an essential facet of RV-induced host cell take over process. Host PRRs which have been implicated to shape innate immune response against RV infection are cell-intrinsic RIG1-like receptors (RLRs) and NOD-like receptors (NLRs) (both of which are activated inside the infected cells), as well as cell-extrinsic Toll-like receptors (TLRs) (which are expressed on cell surfaces and vesicular membranes of uninfected bystander cells including macrophages and dendritic cells) [102]. Unlike the contribution of host PRRs, little is known about the signature RV PAMPs which are engaged in eliciting RV-induced initial IFN response. Because of its inclusion within DLPs and/or viroplasms, the significance of the segmented dsRNA genome to act as a PAMP through involvement of dsRNA-specific PRR TLR3 has been questioned, especially in infected intestinal epithelial cells of suckling mice and human infants where TLR3 expression is poor [103–105]. Within intestinal immune cells such as plasmacytoid dendritic cells (pDCs), however, RV dsRNA can act as PAMP to evoke IFN response, though the involvement of host PRR has remained unaddressed [106–110]. More significant PAMPs in physiological settings of RV infection have been postulated to be RV replication byproducts including RV mRNA species with exposed 5ʹphosphate groups and those with incomplete 5ʹ-O-methylated “cap” structures (a result of inefficient VP3-mediated mRNA-capping) (Figure 3a) [111,112]. Physiologically relevant host PRRs capable of sensing these RV PAMPs and further mounting IFN-response in infected intestinal cells include RLRs such as Retinoic acid-inducible gene 1 (RIG1) and Melanoma differentiation-associated protein 5 (MDA5) (Figure 3a). Detection of RV PAMPs by and subsequent activation of RIG1 and MDA5 induces prion-like oligomerization of the mitochondrial RLR adaptor Mitochondrial antiviral-signaling protein (MAVS) which further recruit TNF receptor-associated factors (TRAFs; TRAF2, TRAF5, TRAF6) and two kinase complexes [Iκβ kinase complex (IKK-α/β/γ) and TANK-binding kinase 1 (TBK1)-IKKε] leading to the activation of Interferon regulatory factor 3 (IRF3) and Nuclear factor kappa-light-chain-enhancer of activated B cells (NF-κB)-dependent transcriptional programme characteristic of initial IFN induction (Figure 3a). MAVS depletion exerted a more severe IFNβ antagonizing effect in RV-infected settings than when RIG1 or MDA5 was depleted alone, suggesting MAVS to act as a common adaptor downstream of both RIG1 and MDA5 [103,104]. Nevertheless, MDA5 overexpression significantly attenuated RV replication through upregulation of many ISGs possibly bypassing the involvement of JAK-STAT pathway [113]. Unlike the initial PAMP sensing machinery, many of the downstream effectors involved in mounting initial IFN response are targeted strategically by RVs, particularly by NSP1 (the IFN antagonist of RV) [114,115]. Non-proteolytic depletion of RIG1 by NSP1 (from both OSU and SA11 strains), possibly effected through NSP1-RIG1 interaction [without involving NSP1 C-terminal (∼170-aa) IRF3-binding domain], has been documented (Figure 3a) [116]. Proteasome-dependent degradation of MAVS has also been reported during later hours of RV infection, possibly via involvement of NSP1 and/or VP3 (Figure 3a) [117–119]. Another proteasome-sensitive substrate was found out to be TRAF2 which was targeted by NSP1 from both IRF3 degrading (simian SA11) and NF-κB inhibiting (porcine OSU, bovine A5-13) RV strains (Figure 3a). TRAF2 degradation by NSP1 was shown to effectively curtail the non-canonical NF-κB pathway induced by exogenous IFN [120]. NSP1 of some RV strains (primarily of porcine and human origin) also inhibits β-TrCP, an F-box protein and essential NF-κB activating factor, by interacting with and/or degrading it in a proteasome-dependent manner through hijacked host cellular Cullin3-RING box 1 (Cul3-Rbx1) E3 ubiquitin ligase machinery (Figure 3a) [121–125]. Notably, NSP1-β-TrCP interaction, rather than β-TrCP degradation, proved to be critical for RV-mediated inhibition of NF-κB [125]. Site-directed mutation studies allowed identification of Casein kinase II (CK-II)-mediated phosphorylation of NSP1 (Serine 480 and 483 in OSU NSP1) to be essential for subsequent β-TrCP interaction (Figure 3a). Mutating CK-II phosphorylation priming site on NSP1 resulted in failure of Cul3 recruitment leading to abrogation of NF-κB inhibition [121,125]. An alternate β-TrCP-independent mechanism by which RV strains block NF-κB function is through prevention of NF-κB p65 nuclear translocation possibly through sequestration of it into viroplasmic puncta (Figure 3a) [124,126,127]. Depending on the viral strain and the host species, NSP1 can also interact with IRFs (IRF3, IRF5, IRF7, and IRF9, but not IRF1) and trigger their proteasomal degradation (Figure 3a) [128]. The first suggestion that NSP1 was involved in modulating the IFN pathway came from a yeast two-hybrid assay in which the interaction between IRF3 and NSP1 was detected [129]. The NSP1-IRF interaction involves the C-terminal domain (the last 326-aa) of NSP1 and the IRF dimerization domains (homo/heterodimers) on IRFs [111,128,130,131]. Interestingly, because of high context-specificity behind NSP1’s IRF degrading property, NSP1-mediated IRF3 inhibition under specific contexts of PRR stimulation extends beyond IRF3 degradation [111]. Abrogation of IRF3 and/or NF-κB-dependent gene transcription during infection with wild type RV strains has been shown to effectively curtail IFN induction cascade beyond early hours.

**Figure 3.**
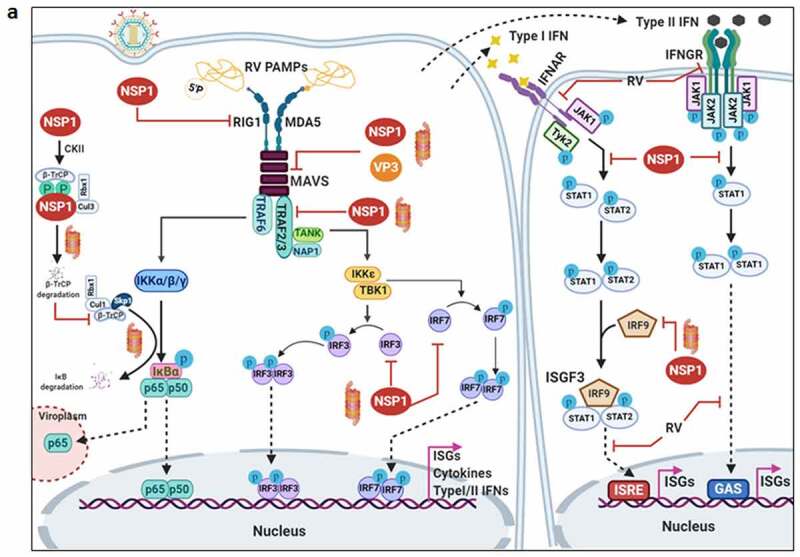
**Evasion of host cellular antiviral responses by RV**. (a) Evasion of host cellular IFN response. Within RV infected cells, RV RNA species with exposed 5ʹ-phosphate groups or with incomplete 5ʹ-O-methylated “cap” structures act as PAMPs and are recognized by host cellular PRRs RIG1 and MDA5. Subsequent oligomerization of the mitochondrial adaptor MAVS forms a platform for recruitment of TRAFs and two kinase complexes IKK-α/β/γ and TBK1-IKKε leading to the activation of IRF3/7 and NF-κB-dependent transcriptional programme (IFNs, ISGs, cytokines). NF-κB contains two subunits p50 and p65. IKK-α/β/γ phosphorylates NF-κB inhibitory protein IκB at the α subunit resulting in its proteasomal degradation by SCF^β-TrCP^ (Skp1-Cullin1-F-box containing protein β-TrCP) E3 ubiquitin ligase complex. Being freed of IκB, p50-p65 heterodimeric NF-κB translocates to nucleus and causes transcriptional activation. RV-NSP1 targets many host proteins of this pathway proteasomally (such as MAVS, β-TrCP, IRF3/7, TRAF2) or non-proteasomally (RIG1) and causes their degradation in a RV strain-dependent manner. RV-VP3 has also been shown to target MAVS for proteasomal degradation. Moreover, at least for β-TrCP degradation, a fostering role of hijacked host cellular Cul3-Rbx1 E3 ubiquitin ligase machinery has been implicated. NSP1-β-TrCP interaction also requires CK-II-directed NSP1 phosphorylation reactions. Some RV strains block NF-κB function through sequestration of NF-κB p65 away from nucleus into viroplasmic puncta. The amplification step of IFN response includes binding of IFNs (type I and type II) to cognate receptors (type I IFN receptor IFNAR and type II IFN receptor IFNGR) to activate JAK-STAT signaling. The phosphorylated forms of STAT1 and STAT2 form a complex called ISGF3 by associating with IRF9. This heterotrimeric complex translocates to the nucleus, binds to IFN-stimulated response elements (ISREs) and trans-activates a series of ISGs of cyto-protective and antiviral nature. Homodimeric phosphorylated STAT1 downstream of IFNGR signaling also trans-activates ISGs by nuclear translocation and binding to Gamma interferon activation site (GAS). RV infection curtails IFN amplification pathway by promoting degradation of IRF9, IFN receptors, and by preventing STAT1 phosphorylation as well as STAT1-STAT2 nuclear translocation. RV-NSP1 is responsible for degradation of IRF9 proteasomally and for inhibition of STAT1 phosphorylation. (b) Evasion of host cellular OAS/RNase L pathway by RV. Within virus infected cells, viral dsRNA population can induce oligomerization-dependent activation of the enzyme OAS which further catalyzes the formation of 2ʹ-5ʹAs. Upon interacting with 2ʹ-5ʹAs, RNase L gets activated through dimerization and triggers cleavage of RNA including the viral RNA species. RV evades the deleterious effects of OAS/RNase L pathway by its structural protein VP3. RV-VP3 has intrinsic 2ʹ, 5ʹ-phosphodiesterase (2ʹ-5ʹPDE) motif through which it disintegrates 2ʹ-5ʹA structures, thereby preventing RNase L activation and viral RNA cleavage. (c) Evasion of innate antiviral impacts of host RNAi machinery by RV. Double stranded RV replication intermediates can potentially be subjected to trimming by host cellular DICER resulting in production of virus‐derived small interfering RNAs (viRNAs). Incorporation of viRNAs into the RISC containing the catalytic effector AGO2 can subsequently target viral RNA population. During early hours of RV-SA11 and RV-A5-13 infection, RV-NSP1 interacts with and ubiquitylates AGO2 leading to proteasomal demise of this catalytic effector. In this respect, RV-NSP1 acts as a putative viral‐suppressor‐of‐RNAi (VSR). In absence of AGO2, siRNA/shRNA-guided RNAi and also potential viRNA-directed RNAi are rendered nonfunctional during early hours of RV infection. Clonal overexpression of AGO2 shows anti-RV effects. (d) Attenuation of host cellular anti-oxidant defense system by RV. Nrf2 is the master transcription factor which deals with cellular redox stress by transcribing anti-oxidant and cyto-protective effectors such as HO-1, NQO1, SOD1. Under unstressed condition, Nrf2 is constantly turned over in a ubiquitin-proteasome-dependent way by cellular Keap1-Rbx1-Cul3 machinery. (Left panel) In RV infected cells, reactive oxygen species (ROS) is induced during early hours leading to Keap1 inhibition and Nrf2 upregulation. Elevated Nrf2, further primed by PKC-mediated phosphorylation, translocates to nucleus and trans-activate stress responsive genes which contain Nrf2-binding motif [anti-oxidant response element (ARE)] in their promoter regions. Quenching ROS by NAC has an antagonizing effect on RV infection. (Right panel) During later hours of RV infection, Nrf2 is expelled out of the nucleus, ubiquitylated by a non-canonical E3 ubiquitin ligase (other than the canonical Keap1-Rbx1-Cul3 machinery) and degraded proteasomally. Levels of HO-1, NQO1, and SOD1 were also reduced. Agonists of Nrf2/ARE pathway, such as Keap1 inhibitors CDDO-Me and RA-839, show potent anti-RV effects. (e) Time-dependent regulation of host cellular apoptotic cell death by RV. (Left panel) During early hours of infection, anti-apoptotic pathways are activated and pro-apoptotic pathways are inhibited for ensuring viral replication. The prime most survival pathway includes activation of PI3K-Akt signaling as a result of interaction of RV-NSP1 with PI3K. Interaction of the chaperone Hsp90 with Akt has an agonistic effect on this pathway. Inhibition of survival pathways through targeting PI3K, phospho-Akt and Hsp90 by LY, 294–002, triciribine and 17-AAG, respectively, sensitized RV replication. Inhibition of pro-apoptotic pathways is multifaceted. One of them is the upregulation of the miRNA population hsa-miR-142-5p by RV-NSP5. Elevated hsa-miR-142-5p sensitizes its targets TGFβR II and SMAD3 leading to attenuation of p38MAPK-ERK1/2-JNK-dependent apoptotic signaling in HT29 cell line. Another strategy is the ubiquitylation and proteasomal degradation of p53 during early infection period. RV-NSP1 plays a pivotal role in this regulation. In absence of p53, transcription of p53-dependent apoptotic genes is prevented. Yet another anti-apoptotic modality in early hours of RV infected cells is the prevention of RV-NSP4 translocation to mitochondria. This is enabled by the ubiquitin-proteasome-dependent demise of the mitochondrial chaperonin Hsp60 which facilitates NSP4 mitochondrial import. A phosphorylation event of Hsp60 carried out by the autophosphorylated and activated form of Src kinase (SrcY416) imparts proteasomal sensitivity to Hsp60. Targeting hsa-miR-142-5p by its anti-miR and Src kinase by a small molecule SKI-I exert anti-RV activity. (Right panel) During late phase of infection, apoptotic pathways are activated and/or de-repressed and outweigh the survival pathways. Intrinsic pathway of apoptosis observed in late hours of RV infected cells is partially dependent on Bax. Subsequent release of cytochrome c into cytosol results in apoptosome formation and activation of executioner caspases. Reduced level of hsa-miR-142-5p results in de-repression of TGFβR II-SMAD3-p38MAPK-ERK1/2-JNK-dependent apoptotic signaling in HT29 cell line. Weakened interaction of p53 with RV-NSP1 stabilizes p53 and causes p53-dependent transcription of pro-apoptotic genes (PUMA, Bax. Bak). In absence of SrcY416, Hsp60 is no longer phosphorylated and therefore escorts NSP4 across mitochondria. NSP4 also positively regulates apoptotic mitochondrial fragmentation by promoting Cdk1-dependent phosphorylation of Drp1 at Serine 616 residue and further recruiting them to mitochondria. NSP4 also promotes mitochondrial translocation of Parkin which reduces mitochondrial fusion by degrading Mfn1. Targeting Drp1 and Cdk1 by respective small molecule inhibitors Mdivi-1 and RO-3306 prevented apoptotic mitochondrial fragmentation and viral progeny release. (f) Subversion of host UPR by RV. Accumulation of misfolded proteins in the ER leads to uncoupling of GRP78 from UPR sensors, resulting in activation of the three branches of UPR-ATF6 pathway, PERK-dependent pathway, and IRE1-based signaling. RV activates two (ATF6 and IRE1) of the three branches of UPR, but limits maturation of the activated UPR pathways. Following RV infection, dissociation of GRP78 from ATF6 (ATF6p90; the transcriptionally inactive fragment) triggers translocation of ATF6 to the Golgi apparatus where it is cleaved and the transcriptionally active fragment ATF6p50 is transported to nucleus to trans-activate UPR elements (CHOP, GADD34, GRP78 and GRP94). Despite the initial activation of ATF6 arm of UPR, RV inhibits further transcription of UPR elements by immobilization of the ATF6p50 fragment into viroplasms. UPR element proteins are also sequestered within viroplasms and further synthesis of them is inhibited by NSP3-induced host translational stasis. Release of PERK from GRP78 leads to homo-dimerization and phosphorylation of PERK; however, RV sequesters p-PERK in the viroplasms inhibiting further activation. Uncoupling of GRP78 from IRE1 leads to homo-dimerization and autophosphorylation of IRE1. Phosphorylated IRE1 (p-IRE1) triggers splicing of xbp1 mRNA (xbp1u) to form a spliced variant (xbp1s). However, further translation of the xbp1s is prevented as a result of general host translational inhibition mediated by RV-NSP3. RV also induces IRE1-independent alternative splicing of xbp1 leading to generation of an exon-skipped splice variant (xbp1es). This event of xbp1 alternative splicing was found to concur with NSP3-mediated PABPC1 nuclear translocation. (g) Evasion of antiviral impacts of SGs and PBs and viroplasmic sequestration of host cellular RBPs. Translational shut off because of phosphorylation of eIF2α is a classical trigger for formation of SGs which accumulate many cellular proteins (see figure). PBs and GW bodies also contain protein conglomerates (see figure). In RV infected cells, eIF2α becomes phosphorylated by PKR, but SG formation is prevented. Punctate PB structures are also absent in infected cells. PB component Pan3 and GW body component AGO2 are degraded proteasomally by RV-NSP1. At later hours, AGO2 relocalizes to viroplasmic niches. Other PB/SG/GW body components are also relocated to different subcellular niches such as nuclear compartment (XRN1, hDcp1, DDX6) or viroplasms (ADAR1, Caprin1, CPEB, eIF2α, PKR, Staufen1, PPM1A, LSM1, PARN, GW182, Caf1) or remained dispersed in cytosol (G3BP1, ZBP1). Moreover, many PB/SG components interact with viroplasmic RV-NSP2 and RV-NSP5. Additionally, many hnRNPs and ARE-BPs are re-located from nucleus to cytosol, get sequestered to viroplasms, and interact with RV-NSP2 and RV-NSP5 within RV infected cells. Many relocated RBPs are also absorbed by the copious viral transcripts

**Figure 3.**
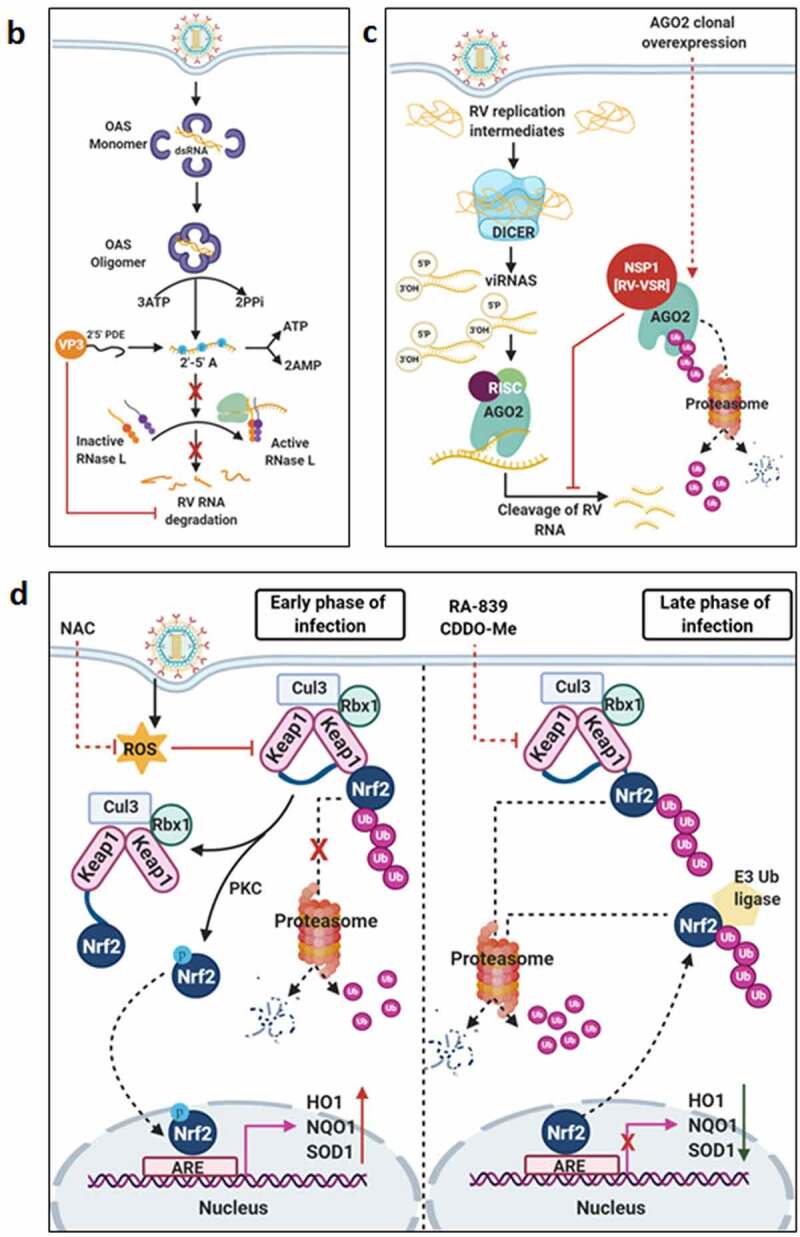
Continued

**Figure 3.**
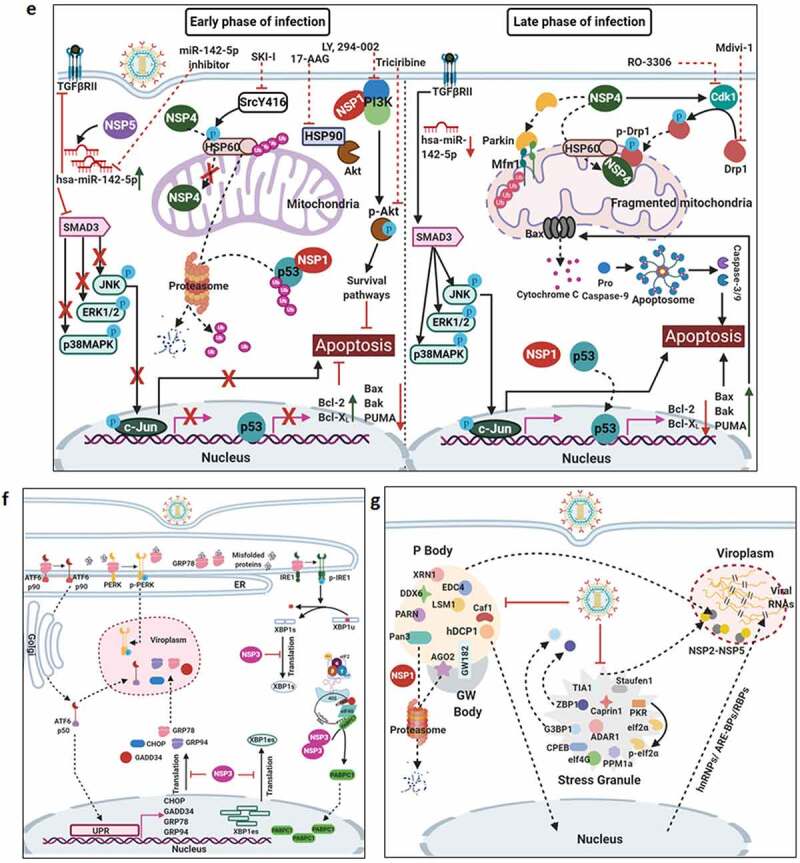
Continued

RVs also dampen IFN response at the signal amplification stage. Core to the signal amplification-based transcriptional reprogramming is phosphorylation of STAT1 and STAT2, nuclear translocation and association of the STAT1-STAT2 heterodimer with IRF9 forming the IFN stimulated gene factor 3 (ISGF3) complex (Figure 3a) [132]. RVs have been shown to block activation of STATs by precluding STAT1 phosphorylation (within infected as well as bystander cells) and inhibiting STAT1 and STAT2 nuclear translocation (within RV-infected cells) (Figure 3a) [126,127,133]. Relative interdependence of these two processes and the viral trigger as well as the host contributors behind such STAT antagonism have been poorly addressed. At least for STAT1 phosphorylation inhibition, a role of NSP1 has been implicated (Figure 3a) [133]. Moreover, degradation of type I, II, and III IFN receptors within RV infected cells through lysosomal-proteasomal pathway has also been evidenced recently (Figure 3a) (Figure 3a) [134].

Interestingly, two principal lines of evidence vouch for the existence of stringent host range restriction of RVs especially in context of establishing efficient infection in suckling mice model. Firstly, homologous RV strains (murine strain EW) effectively infect suckling mice and are largely insensitive to antiviral effects of type I and type II IFN primarily because of their ability to curtail IFN-mediated antiviral responses. Secondly, heterologous RV strains (simian strain RRV, SA11) have poor replication potential in mice model but can reach to high titers upon inhibition of the IFN signaling (combined knockouts of the type I and II IFN receptors/STAT1 knock out). Implication of RV-NSP1 in shaping the IFN response in a host range restricted manner has been well established [135–137]. To add complexity, certain heterologous strains (such as bovine strain NCDV and porcine strain OSU) cannot achieve high replication efficiency even in IFN receptor or STAT1 deficient mice because of their entry restriction (VP4-based) [136,137]. Nonetheless, in cultured human intestinal epithelial cell line (Caco2 and HT-29), a 3-day pretreatment regime of IFNs caused one log reduction of viral (human and simian strains) titer [18,115,138]. A few reports also advocated for IFN-based anti-rotaviral effects in cell culture [138–140], mice model [139] and also in human intestinal organoid model [140,141].

### Countering the 2ʹ, 5ʹ-oligoadenylate synthetase/RNase L pathway

Apart from dampening IFN response, RVs have been shown to evade potential deleterious effects of OAS/RNase L pathway. The prime most antiviral effector belonging to this pathway is the enzyme RNase L which upon activation cleaves viral as well as cellular RNAs leading to cellular demise. Activation of RNase L is mediated by 2ʹ, 5ʹ-oligoadenylates (2ʹ-5ʹAs) which in turn are synthesized by activated OAS downstream of dsRNA recognition (Figure 3b). Interestingly, C-terminal domain of RV VP3 has been shown to antagonize the antiviral activity of RNase L by cleaving the 2′-5′-phosphodiester bond of the oligoadenylates via its 2ʹ, 5ʹ-phosphodiesterase motif (Figure 3b) [142–146].

### Evading the antiviral effects of RNA interference

RNAi is an evolutionary ancient antiviral innate immune response in plants, nematodes, and arthropods. RNAi functionality includes dicing of dsRNAs of exogenous (such as viral) or endogenous origin by RNase III‐like endonuclease DICER into trimmed RNA duplexes and further processing of those duplexes within the RNA‐induced silencing complex (RISC) to finally result in cleavage or translation repression of the mRNA target (Figure 3c). As a part of the counter‐defensive measures, viruses have evolved to produce virulence determinants called viral‐suppressors‐of‐RNAi (VSRs) [147–149]. Interestingly, in spite of functional preponderance of IFN signaling‐based immunity over RNAi in somatic cells of higher vertebrates, retention of the antiviral nature of RNAi has been advocated for quite a few times in mammalian cells [150,151]. RV dsRNAs are usually encaged within DLPs and viroplasms during the entire span of the viral life cycle. Oozing of rotaviral dsRNA intermediates, however, has been observed in unmasked cytosolic environment [152,153], which may potentially evoke RNAi‐based surveillance mechanism. Interestingly, RV infection has been found to cripple small interfering RNA (siRNA)/short hairpin RNA (shRNA)-directed RNAi functionality [(but not of functionality of microRNA (miRNA)] during early hours (2–6 hpi) of infection. This is enabled by triggering RV-NSP1-mediated ubiquitylation and subsequent proteasomal degradation of Argonaute2 (AGO2) which is the prime catalytic effector of siRNA‐mediated RNAi within RISC of mammalian cells (Figure 3c) [154]. Clonal overexpression and silencing of AGO2 had a respective antagonistic and augmentative effect on RV infection, further corroborating antiviral importance of AGO2 [154–156]. Of interest, overexpression of AGO1 or the catalytically dead mutant of AGO2 could not curtail RV infection, emphasizing the exclusivity of slicing-competent AGO2 in exerting anti-RV effects [154]. Notably, reinstatement of RNAi functionality beyond 6 hpi has also been found to coincide with reduced NSP1-AGO2 association and diminished AGO2 K48-linked ubiquitylation. From the perspective of rotaviral physiology, however, crippling siRNA‐mediated RNAi during early hours of infection may potentially be advantageous as the viral genome would not be susceptible to processing by cellular RNAi machinery in this vulnerable phase when viroplasmic sequestration of dsRNAs is not absolute (Figure 3c) [154]. Strikingly, RV-mediated perturbation of RNAi competency poses an apparent contrast with many previous reports where functionality of siRNA/shRNA-guided RNAi pathway has been shown to be retained during infection [157–160]. Notably, in most of these experiments, RNAi functionality was assessed beyond 6‐hpi. Moreover, efficient knocking down of antiviral host targets, especially those involved in viral entry and early viral life cycle events, prior to infection suggests viral entry and subsequent life cycle stages to be impaired. Insufficient viral load, therefore, may also hinder AGO2 degradation, explaining retention of RNAi functionality. Corroborations along this line of argument await further experimentation. Moreover, based on the report of insensitivity of AGO2 to RRV during early hours of infection [155], follow-up studies are important to evaluate the status of time point-dependent RNAi functionality in response to different RV strains.

### The dynamics between rotavirus infection and the host cellular anti-oxidant defense system

Adaptive cellular responses to oxidative and electrophilic stress are usually taken care of by an anti-oxidant defense system, core to which lies the redox-responsive transcription factor Nuclear factor erythroid-derived-2-like 2 (Nrf2) and Nrf2-driven transcriptional cascade (Figure 3d). As a part of the avoidance of cellular stress-response pathways, deregulation of host redox balance and redox stress-sensitive Nrf2 anti-oxidant defense have been reported for many viruses [161,162]. Upsurge of oxidative stress during initial hours of rotaviral infection has been cited a few times [163,164]. Moreover, downregulation of host anti-oxidant repertoire has been evidenced in animal model studies of RV-induced gastroenteritis [165,166]. Supportive findings also reported anti-oxidative cellular environment, generated thorough pharmacological intervention [by using N-acetyl-L-cysteine (NAC)], to exert potent inhibitory effects on RV infection *in vitro* (Figure 3d) [167], in mice model of infection [168] as well as in clinical patients suffering from RV-induced diarrhea [169]. Consistently, stabilization of Nrf2 leading to activation of Nrf2-governed transcriptional network by a recently discovered small molecule RA-839 significantly reduced RV RNA transcripts, protein expression, viroplasm formation, viral titer and RV-mediated host cellular cytopathy, emphasizing the importance of Nrf2-dependent signaling pathway as a druggable anti-rotaviral host determinant (Figure 3d) [170]. Moreover, anti-rotaviral effects of RA-839 were also mimicked by CDDO-Me and Hemin, two classical pharmacological activators of Nrf2/ARE pathway (Figure 3d) [170]. Subsequent mechanistic studies revealed Nrf2 protein levels to decline sharply with progression of RV infection beyond an initial upsurge. Moreover, Nrf2 decrease as a whole was found to be accompanied by active nuclear vacuity of Nrf2, resulting in lowered expression of stress-responsive Nrf2 target genes Heme oxygenase-1 (HO-1), NAD(P)H Quinone Dehydrogenase 1 (NQO1) and Superoxide dismutase 1 (SOD1) both in presence and absence of Nrf2-driven transcriptional inducers. Initial induction of Nrf2 concurred with RV-induced early burst of oxidative stress and therefore was sensitive to treatments with anti-oxidants. Reduction of Nrf2 levels beyond initial hours, however, was found to be independent of cellular redox status and canonical Nrf2 turn-over pathway but dependent on ubiquitin-proteasome system through a non-canonical E3 ubiquitin ligase (Figure 3d) [171].

### Modulation of the cell death pathways

There exists an intricately tuned interplay between virus infection and host cell death pathways to ensure usurpation of host resources for viral propagation before the onset of cellular demise [172]. Not surprisingly, reports of RV infection to modulate apoptotic mode of programmed cell death pathways reiterate the same- an infection time point-dependent bimodal regulation of apoptosis where viral subversive strategies have been shown to prevent apoptotic demise of host cells during early hours of infection only to be reoriented at later phase for apoptotic dissemination of viral progeny. Hall marks of apoptosis have been evidenced in late-phase RV-infected cells along with the observation of mitochondrial membrane depolarization, cytochrome c release into cytosol, caspase 3 activation and cleavage of poly(ADP-ribose) polymerase, suggesting activation of intrinsic apoptotic pathway. Notably, RV-induced apoptosis was found to be partially sensitive to RNAi-mediated Bcl-2-associated X protein (Bax) silencing and BAPTA-AM-mediated Ca^2+^-chelation, indicating involvement of the proapoptotic B-cell lymphoma 2 (Bcl-2) family member Bax and elevated cytosolic Ca^2+^ levels in host cellular apoptosis [173,174]. Indeed, a decrease of antiapoptotic Bcl-2 protein and a concomitant increase in the Bax/Bcl-2 ratio leading to Bax activation have been observed in RV-infected cells (Figure 3e) [174]. A more direct proapoptotic role of NSP4, independent of Bax activation and Ca^2+^-elevation, was further reported where NSP4 was shown to translocate to mitochondria to trigger intrinsic apoptotic cascade [175]. A recent report also highlighted the crucial role of NSP4 in being involved in increasing the fission-active pool of Ser616 phospho Dynamin related protein 1 (Ser616 pDrp1) through augmented activity of cyclin-dependent kinase 1(Cdk1) and further in recruiting them to mitochondria for triggering Drp1-dependent mitochondrial fragmentation (Figure 3e) [176]. In addition to its positive role in mitochondrial fission, Drp1 also resulted in mitochondrial translocation of E3‐ubiquitin ligase Parkin leading to degradation of mitochondrial fusion protein Mitofusin 1 (Mfn1) during RV infection, thereby aggravating the disrupted mitochondrial morphology (Figure 3e). This suggests an efficient strategy utilized by RV to harness programmed cell death to mitochondrial dynamics resulting in apoptotic mitochondrial fission and subsequent dissemination of viral progeny. Consistently, Drp1 inhibition (by Mdivi-1) or prevention of Ser616 phosphorylation of Drp1 (by inhibiting Drp1-phosphorylating kinase Cdk1 via RO-3306) has been shown to cause marked reduction in RV‐NSP4‐induced intrinsic apoptotic signaling and subsequent apoptotic dissemination of rotaviral progeny (Figure 3e) [176].

Very interestingly, a counter-intuitive strategy exists in RV-infected cells for prevention of NSP4-induced host cellular apoptosis during early hours of infection. A crucial positive co-relationship was evidenced between import of NSP4 into mitochondria and the mitochondrial chaperonin Hsp60 where Hsp60 was found to facilitate refolding of denatured NSP4 after the latter gets translocated to mitochondria. During early hours of infection, mitochondrial Hsp60 underwent tyrosine phosphorylation by activated Src kinase and therefore became vulnerable to ubiquitin-proteasome-dependent degradation. This transient degradation of Hsp60 during early hours of RV infection has been speculated to prevent premature apoptosis by delaying mitochondrial import of NSP4. Indeed, Src kinase inhibition (by a small molecule SKI-I) resulted in reduced viral titer owing to NSP4-induced premature abortive apoptosis of host cells (Figure 3e) [175]. Moreover, Drp1-dependent mitochondrial fragmentation triggered in presence of NSP4 was only observed at late phase of infection and therefore temporally concurred with apoptotic dissipation of viral progeny [176].

An important contribution of NSP1 in host cellular apoptosis regulation has further been highlighted where NSP1 was shown to facilitate evasion of premature apoptosis at least by two mutually exclusive mechanisms. A direct interaction of NSP1 with the PI3K regulatory subunit p85 and subsequent activation of the cell survival signaling through PI3K/Akt has been evidenced during early hours of RV infection (Figure 3e) [177,178]. Interestingly, unlike the isogenic wild-type RV strain A5-13, the NSP1 mutant strain A5-16 could not trigger robust PI3K/Akt activation, leading to early induction of apoptosis in A5-16 infected cells [177]. This partially explains slower growth rate and low progeny yield of A5-16 compared to A5-13 under identical infection conditions. Consistently, inhibition of PI3K (by a small molecule LY, 294–002) and phospho-Akt (by triciribine) significantly curbed growth of the RV strain A5-13 (Figure 3e) [177]. PI3K/Akt activation was also found to be sensitive to Hsp90 inhibition as a result of reduced interaction between Hsp90 and Akt [179]. Not surprisingly, treatment of RV-infected cells with 17-AAG, a highly specific Hsp90 inhibitor, resulted in significantly reduced RV gene expressions and RV titers (Figure 3e) [179]. Concomitant inhibition of p53-dependent pro-apoptotic signaling has also been documented in presence of NSP1 during early hours of infection (Figure 3e) [180]. Detailed mechanistic study unraveled NSP1 to directly interact with p53 resulting in its ubiquitylation [via NSP1’s N-terminal Really interesting new gene (RING) domain with putative ubiquitin ligase activity] and proteasomal degradation. Degradation of p53 during initial stages of infection inhibited apoptosis, as the proapoptotic genes p53 upregulated modulator of apoptosis (PUMA) and Bax were downregulated (Figure 3e). NSP1 mutant RV strain A5-16 could not degrade p53, resulting in Bax activation and PUMA upregulation during early infection phase [180].

During later phase of infection, however, NSP1 wild type RV strains down-regulate PI3K/Akt-dependent pro-survival signaling and initiate p53-dependent proapoptotic signaling (enabled by weakened NSP1-p53 interaction and p53 stabilization) (Figure 3e) [180]. Therefore, infection time point-dependent intricate modulation of anti-apoptotic and pro-apoptotic pathways regulated by a finely tuned interplay between NSP1 and NSP4 ensures successful RV perpetuation.

A recent study also unfolded a novel role of RV viroplasmic protein NSP5 in preventing premature apoptosis by suppressing non-canonical Transforming growth factor-β (TGFβ) signaling in the microsatellite stable colon carcinoma cell line HT29 [where canonical TGFβ signaling is nonexistent due to a nonsense mutation in Mothers against decapentaplegic homolog 4 (SMAD4)]. TGFβ up-regulation, as has been observed in RV-infected cells, can induce apoptosis downstream of activated Tumor necrosis factor receptor-associated factor 6 (TRAF6)-Transforming growth factor-β-activated kinase 1 (TAK1)-p38 Mitogen-Activated Protein Kinase (p38MAPK)/c-Jun N-terminal kinase (JNK) pathway (Figure 3e). Interestingly, NSP5-mediated up-regulation of the miRNA hsa-miR-142-5p has been shown to directly target Transforming growth factor-β receptor II (TGFβR II) and Mothers against decapentaplegic homolog 3 (SMAD3) leading to attenuation of non-canonical TGFβ signaling. Consistently, exogenous expression of hsa-miR-142-5p inhibitor resulted in a significant reduction of viral titer most likely by triggering TGF-β induced premature apoptosis (Figure 3e) [181].

Unlike regulation of apoptosis, reports on non-apoptotic mode of programmed cell death pathways (such as pyroptosis and necroptosis) are very limited in the context of RV infection. A recent study demonstrated possible antiviral implications of pyroptosis downstream of a cross-talk between RV and NLR inflammasome within infected cells [153]. The inflammasome is a complex of cytosolic proteins which aggregate to mediate proteolytic processing of pro-IL-1β and pro-IL-18 and the pore-forming protein gasdermin D, leading to pyroptosis that liberates biologically active IL-1β and IL-18 from the cell [182]. Mechanistically, the NLR Nlrp9b was found to recognize short dsRNA stretches (of RV) in an RNA helicase Dhx9-dependent way to form inflammasome complexes with the adaptor proteins Apoptosis-associated speck-like protein containing a CARD (Asc) and caspase-1 leading to IL-18 maturation and gasdermin D-induced pyroptosis. Conditional depletion of Nlrp9b or other inflammasome components in the intestine *in vivo* resulted in enhanced susceptibility of mice to RV replication [153].

## Evasion of unfolded protein response

The unfolded protein response (UPR) is a cellular homeostatic mechanism which ensures coping up with the stress-induced accumulation of misfolded proteins within ER by reducing global translation at the expense of synthesis of selective transcription factors which further trans-activate UPR-responsive genes. Failure to effectively negotiate with the misfolded protein cargo sets in signaling cascade leading to the cellular demise. Core to initiating this synchronized response are three ER membrane sensors: the endoribonuclease inositol-requiring enzyme 1 (IRE1), activating transcription factor 6 (ATF6), and PKR-like ER kinase (PERK) (Figure 3f) [183]. Viral hijacking of host cells often mobilizes ER stress-mediated UPR which may heavily influence viral replication [184]. Interestingly, RV infection has been found to activate two of the three arms of UPR (IRE1 and ATF6) by multiple viral proteins; however, canonical maturation of the pathways become hindered at the translational level because of NSP3-mediated host cellular translational stasis (Figure 3f) [185]. Some other key effectors of UPR such as PERK, C/EBP homologous protein (CHOP), and Growth arrest and DNA damage-inducible protein (GADD34) have also been observed to get relocalized to/or near viroplasms, further restricting UPR maturation (Figure 3f) [186]. Thus, even though host cells trigger UPR in response to RV infection, RV has developed evasive strategies for avoiding the potential deleterious effects of this antiviral host response.

Interestingly, apart from the alternative splicing of X-box binding protein 1 (xbp1) RNA in cytosol downstream of RV-mediated IRE1 activation, a recent report also demonstrated a RV strain–specific exon skipping phenomenon (lacking exon 4) of xbp1 RNA independent of IRE1 [187]. Functional dissection through rigorous reverse genetics approach only enabled to reveal a concurrency of this exon skipping with Poly(A) binding protein cytoplasmic 1 (PABPC1) nuclear re-localization by eukaryotic Initiation Factor 4 G (eIF4G)-binding domain of NSP3 (Figure 3f) (the latter event is described in the following sections). Although the exact functional significance of RV-induced xbp1 exon skipping on regulating host innate immune response has remained unaddressed, speculations regarding a global change in the splicing landscape within RV-infected cells have been made [187].

### Hindering canonical formation of processing bodies and stress granules through sequestration at atypical niches

Eukaryotic cells possess different cytoplasmic mRNA-protein inclusion foci which are endowed with the property of regulating gene expression, metabolic homeostasis, and also of eliciting response feedback against stress-induced global translational arrest. These membrane-less, dynamic, cytoplasmic granules include stress granules (SGs; which contain translation initiation-stalled mRNAs/translation initiation factors), processing bodies (PBs; with translationally repressed and potentially decaying mRNA along with the mRNA decay factors), and GW182 bodies (GW-bodies; involved in nonsense-mediated decay and microRNA-mediated silencing). Partitioning of eukaryotic mRNA between polysomes, SGs, and PBs/GW-bodies has been reported to dictate the fate of the mRNA population by governing the rate of mRNA translation and mRNA repression/degradation/decay which further regulate gene expression [188]. Interestingly, RVs have been shown to evade potential deleterious effects of this eukaryotic mRNA surveillance machinery, thereby ensuring unrestricted translation of viral mRNAs on cellular polysomes. Core to this evasion strategy has been RV-mediated active prevention of mRNA granule formation, even in the presence of exogenous stressor such as sodium arsenite, coupled to the re-organization of the granular components to different subcellular locations (Figure 3g) [155,156,189–191]. Though there are contrasting reports on altered subcellular niches of SG/PB/GW-body components in RV-infected cells, absence of SG-specific (evidenced by visualizing SG components such as G3BP1, TIA1, ZBP1 staining), PB-specific (evidenced by visualizing PB components Pan3, hDCP1a, XRN1, DDX6, LSM1), and GW-body-specific (evidenced by visualizing GW182, AGO2) puncta have been demonstrated unequivocally (Figure 3g) [155,189–191]. Several molecular mechanisms have been put forward to explain RV-induced disruption of SGs/PBs/GW-bodies. NSP1-mediated proteasomal degradation of a nucleating PB component Pan3 partially explains PB disruption in RV-infected cells (Figure 3g). However, proteasome-insensitive Pan3 in cells infected with NSP1 mutant A5-16 also failed to form PB puncta [189]. Similarly, Ras-GTPase-activating protein (SH3 domain) binding protein 1 (G3BP1) (a SG marker) and GW182 (a GW-body marker), whole cell levels of which remained unperturbed during RV infection, also showed no aggregation in RV-infected cells, suggesting involvement of additional mechanisms [155,156,189]. Interestingly, in RV-infected cells, most of the SG/PB/GW-body components have been shown to alter their sub-cellular niches including non-canonical nucleus-cytoplasmic distribution and viroplasmic sequestration which might rationalize their exclusion from the canonical aggregates (Figure 3g) [155]. Nucleus to cytoplasmic redistribution of many RNA binding proteins (RBPs), some of which are also components of SGs/PBs, and their interaction with viral mRNAs, suggest a possible sponging effect of the copious viral transcripts behind such redistribution (Figure 3g) [156,192]. Moreover, with selective exclusion of a few SG (G3BP1 and ZBP1) and PB components (DDX6, EDC4, and Pan3), many other SG/PB/GW-body constituents get sequestered within (or around) viroplasms and also get engaged in direct, RNA-independent interaction with viroplasmic RV proteins (NSP2, NSP5) to form remodeled/atypical aggregates within RV-infected cells (Figure 3g) [155]. Importantly, RNAi-mediated silencing of many SG/PB/GW-body proteins resulted in increased viroplasmic protein (NSP2, NSP5, VP6) expressions and enhanced infectious progeny yield, indicating antiviral importance of these host cellular determinants. Consistently, ectopic overexpression of some of these potentially antiviral host proteins (G3BP1, Caprin, Dcp1a, Caf1) resulted in reduced rotaviral titer [155].

## Dethroning the host: Usurping host machineries to facilitate viral life cycle events

### Host machineries usurped for viral translation

Preferential synthesis of viral proteins by hijacking host cellular translation machineries in a cellular environment, which is otherwise suppressive for host protein translation is the characteristic feature of RV protein synthesis. Global translational shut off during RV infection has been shown to occur by at least three mechanisms – inhibition of translation initiation by Protein kinase R (PKR)-mediated eukaryotic initiation factor 2α (eIF2α) phosphorylation [152,191], re-localization of cytoplasmic Poly(A)-binding protein PABPC1 to nucleus leading to stalled nucleo-cytoplasmic shuttling of cellular messages [193], and polysomal occupancy by viral messages through outcompeting cellular transcripts.

GTP hydrolysis of eIF2 and its subsequent regeneration through the activity of a guanine exchange factor eIF2B is extremely essential for translation initiation. When phosphorylated at the α subunit, however, eIF2 sequesters eIF2B in an inactive peIF2–eIF2B complex, thereby sensitizing translation initiation (Figure 4a) [194]. Interestingly, RV infection induces dsRNA-dependent activation of PKR which further phosphorylates eIF2α leading to inhibition of host translation initiation (Figure 4a) [152,191]. Indeed, RNAi-mediated PKR silencing or PKR knock-out condition prevented RV-induced eIF2α phosphorylation and restored host protein synthesis [152]. Interestingly, translation of viral messages was shown to be unaffected in presence of peIF2α, justifying why cellular transcripts are outcompeted by viral messages during infection [152]. Moreover, peIF2α is a classical trigger for formation of G3BP1-dependent SGs where stalled translation initiation factors and mRNAs are segregated temporarily. RVs have been shown to actively prevent SG formation, thereby preventing viral RNAs from being targeted within SGs [155,191].

**Figure 4.**
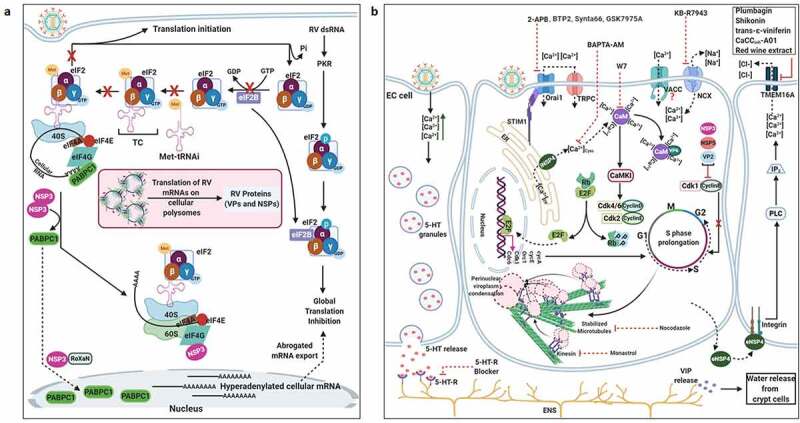
**Usurpation of host machineries by RV**. (a) Preferential synthesis of viral proteins at the expense with host protein translation. The eukaryotic initiation factor 2 (eIF2) is needed to form the ternary complex (TC) [consisting of eIF2 (α, β, and γ subunits), Met-tRNAi (initiator tRNA carrying methionine), and GTP], which in turn escorts the initiator Met-tRNAi to the P site of the 40S ribosomal subunit, enabling translation initiation. The delivery of the initiating amino acid requires GTP hydrolysis. The guanine exchange factor eIF2B is needed for the conversion of eIF2-GDP to eIF2-GTP for translation to continue. In response to RV infection, the α subunit of eIF2 becomes phosphorylated by the viral dsRNA-activated PKR. The p-eIF2α sequesters eIF2B in an inhibitory complex, leading to prevention of ternary complex formation and global translation inhibition. Efficient translation of eukaryotic mRNA also requires mRNA circularization through interaction between eIF4E (cap-binding protein at the 5ʹ termini) and PABPC1 [Poly(A) tail binding protein at the 3ʹ termini] via an intermediate scaffold eIF4G. During RV infection, NSP3 interacts with eIF4G thereby evicting PABPC1 from PABPC1-eIF4G complex; NSP3 also promotes nuclear re-localization of PABPC1 by interacting with a cellular protein RoXaN. Nuclear aggregation of PABPC1 causes accumulation and hyperadenylation of poly(A)-containing mRNAs because of abrogated nucleus-to-cytoplasmic mRNA export. In times of host translational stasis, rotaviral transcripts are translated on host cellular polysomes to produce viral proteins. (b) Regulation of host calcium metabolism and cell cycle progression by RV. Within virus infected cells, RV-NSP4 acts as a viroporin on ER membrane and releases Ca^2+^ from ER. Loss of ER Ca^2+^ activates ER-resident Ca^2+^-sensor STIM1 which translocate to plasma membrane to further activate calcium channels such as Orai1 and TRPC. Other calcium channels such as sodium/calcium exchanger (NCX) and voltage-activated Ca^2+^ channels (VACC) also regulate Ca^2+^ homeostasis; contribution of VACC, however, has been questioned. Several Ca^2+^ channel blockers, which sensitize NSP4-mediated calcium entry, are shown. Among the signaling cascades activated downstream of increased cytosolic Ca^2+^ are the Ca^2+^/CaM/CaMKI/Cyclin-Cdk/Rb/E2F signaling axis to facilitate G1-to-S phase transition of host cells (and the autophagic signaling to aid in outer capsid assembly of progeny virions; omitted here for clarity; shown in detail later). RV-VP6 also interacts directly with CaM in a Ca^2+^-dependent way. Ca^2+^ chelation by BAPTA-AM has a negative impact on Ca^2+^-induced signaling pathways in RV infected cells and reduces RV progeny yield. Moreover, targeting CaM by a small molecule inhibitor W7 also abrogated CaM/CaMKI/Cyclin-Cdk/Rb/E2F signaling axis and antagonized RV progeny production. Besides G1-to-S phase transition, prolonged intra-S phase retention of RV-infected cells is also enabled by a blockade of entry into M phase by cell cycle arrest at S-G2 check point. This is accomplished by the depletion of cyclin B1 and subsequent inhibition of the Cdk1-cyclin B1 complex. RV proteins NSP3, NSP5, and VP2 regulate cell cycle arrest at S-G2 check point. Inhibition of mitotic entry ensures preservation of hyperacetylated and stabilized microtubular structures which along with the kinesin motor protein Eg5 facilitate viroplasmic condensation and peri-nuclear relocalization. Targeting microtubule and Eg5 kinesin by small molecules nocodazole and monastrol, respectively, impairs viroplasm dynamics. Secreted extracellular NSP4 binds to integrins on neighboring uninfected cells and activates PLC-IP3-dependent signaling cascade to elevate intracellular Ca^2+^ which in turn triggers Ca^2+^-dependent Cl^−^ secretion through calcium-activated chloride channels (CaCC) such as TMEM16A. Blockers of CaCC, which prevent Ca^2+^-dependent Cl^−^ secretion, are also shown. Increased cytosolic Ca^2+^ in EC cells cause granular release of 5-HT which stimulates ENS to release VIP from nerve endings adjacent to crypt cells. VIP triggers increased water and Cl^−^ secretion from crypt cells. 5-HT receptor blockers and VIP receptor antagonists attenuate RV-induced diarrheagenic response. (c) Usurping host cellular lipid droplets for rotaviral viroplasm assembly. LD biogenesis occurs from within ER and precedes sequential enzymatic reactions. Synthesis of fatty acid palmitate from acetyl-CoA and malonyl-CoA requires the FASN complex. The enzyme ACC-1 catalyzes the conversion of acetyl-CoA into malonyl-CoA. Alternatively, the enzyme ACSL converts the fatty acids which are transported across the plasma membrane from the extracellular source into corresponding fatty acyl-CoA esters. Subsequent synthesis of triacylglycerols (TAG) from fatty acid palmitate and sterol esters (SE) from cholesterol requires the ER-localized enzymes DGAT1/DGAT2, and ACAT1/ACAT2, respectively. These neural lipids accumulate within the lipid bilayers of the ER and get incorporated within the nascent LDs which bud from the outer leaflet of the ER into the cytosol. LDs gradually acquire signature protein markers (such as ADRP and Perilipins). In RV infected cells, LDs act as scaffolds for viroplasm formation and maturation. The nucleation step of viroplasms involves two non-structural RV proteins NSP2 and NSP5. Autophosphorylated and cytoplasmically dispersed NSP2 (dNSP2) interacts with hypophosphorylated NSP5 to finally result in viroplasm formation through a series of phosphorylation reactions on NSP5 (thereby forming hyperphosphorylated NSP5) by NSP2 kinase activity and a host cellular kinase CK1α. Within RV infected cells, ADRP, Perilipin A, and CK1α co-localize with viroplasmic NSP2 (vNSP2) and NSP5. Different enzymes of neutral lipid biosynthesis pathway can be targeted with small molecules to result in inhibition of viroplasm formation/maturation and reduced RV progeny production; ACAT1/2 inhibition by CI-976, PHB; ACSL inhibition by Triascin C; ACC-1 inhibition by TOFA; FASN inhibition by C75; DGAT1/2 inhibition by Betulinic acid, A922500. Similarly, silencing CK1α by siRNA leads to impairment of viroplasm dynamicity and RV progeny yield. (d) Interfering with the host cellular nucleotide biosynthesis pathway sensitizes RV infection. Purine biosynthesis *de novo* (coded in sky blue color) requires a series of enzymatic reactions in which one of the steps is oxidation of inosinate (IMP) to form xanthylate (XMP). This reaction is catalyzed by the enzyme IMP dehydrogenase (IMPDH) which has two isoforms IMPDH1 and IMPDH2. MPA inhibits *de novo* purine biosynthesis by targeting IMPDH2 and exerts anti-RV effects. Similarly, pyrimidine biosynthesis *de novo* (coded in light green color) includes two intermediate steps: oxidation of dihydroorotate to orotate by DHODH and formation of uridylate (UMP) from orotidylate (OMP) by ODcase. Targeting DHODH by small molecules such as BQR, LFM and ODcase by 6-AU impairs *de novo* pyrimidine biosynthesis and exerts anti-RV effects. Anti-RV impacts of gemcitabine depends on inhibition of salvage pathway of pyrimidine biosynthesis (coded in pink color). (e) Usurping a non-canonical DDR signaling by RV to regulate viroplasm dynamicity. (Lower panel) In RV infected cells, MRN-ATM-Chk2 branch of DDR is activated in absence of nuclear DNA damage by an unknown RV-induced mechanism. Formation of γ-H2AX-positive nuclear foci is also prevented. MRN components, ATM, and Chk2 are translocated from nucleus to cytopolasm and accumulate within viroplasmic niches to regulate viroplasm dynamicity. Targeting Mre11, ATM, and Chk2 by small molecule inhibitors Mirin, KU55933, and BML-277, respectively, antagonize RV replication. KU55933 and BML-277 prevent viroplasm condensation and maturation. (Upper panel) However, enforcing DNA damage externally by etoposide or by disintegrating nuclear Cohesin complex (because of loss of STAG2) causes nuclear induction of γ-H2AX and cytosolic accumulation of DNA leading to the activation of cGAS-cGAMP-STING-TBK1-IRF3 signaling. IRF3-dependent IFN synthesis and subsequent amplification of JAK-STAT-mediated antiviral IFN response lead to a RV-refractory host cellular state; cGAMP (2′3′-Cyclic GMP-AMP) (f) Co-opting host cellular ATP synthase for rotaviral life cycle. Three subunits of mitochondrial ATP synthase holoenzyme (ATP5B, ATP5A1, ATP5O) are re-located to viroplasms and interact with 3ʹ UTR consensus of RV RNAs (5ʹ-UGUGACC-3ʹ). Potential involvement of an intermediate viral protein (such as VP1) has been speculated to facilitate the ATP synthase-3ʹ UTR association. Chemical inhibitors targeting ATP synthase such as Isoapoptolidin, Venturicidin, and BDM antagonize RV progeny yield. (g) Exploiting host cellular autophagic machinery for rotaviral morphogenesis. Induction of autophagic signaling occurs in RV infected cells by two mutually exclusive triggers. One of the triggers includes two miRNAs, miR-99b and let-7 g, as the pivotal regulatory components. RV infection increases miR-99b population and decreases let-7 g. Increased miR-99b suppresses mTOR which is a direct target of it. Moreover, reduced let-7 g increases its target TSC1 leading to stabilization of TSC1-TSC2 complex. Because of the GTPase activity of TSC2 for Rheb-GTP, TSC1-TSC2 stabilization results in attenuated levels of Rheb-GTP pool. Rheb-GTP being a positive regulator of mTOR, RV-mediated Rheb-GTP depletion antagonizes mTOR. The other stimulus initiates with the cytosolic release of Ca^2+^ through the NSP4 viroporin activity on ER membrane, followed by the sequential activation of CaMKK-β and AMPK. AMPK directs TSC1-TSC2 stabilization which finally culminates in mTOR inhibition. Overall mTOR restriction causes de-repression of ULK1 complex which subsequently forms phagophore through Beclin1 complex activation and LC3 II lipidation. However, autophagosomes are prevented from lysosomal targeting in RV infected cells; instead, they are utilized for carrying the RV proteins NSP4 and VP7 coming out with the ER-derived COP-II vesicles to maturing progeny virions within viroplasms, thereby aiding in outer capsid assembly. Host regulators that can be targeted for inhibiting autophagy and restricting RV infection are shown; AMPK, CaMKK-β, Vps34 and miR-99b inhibition by Dorsomorphin, STO-609, 3-MA, and anti-miR-99b, respectively; let-7 g elevation by let-7 g mimic

**Figure 4.**
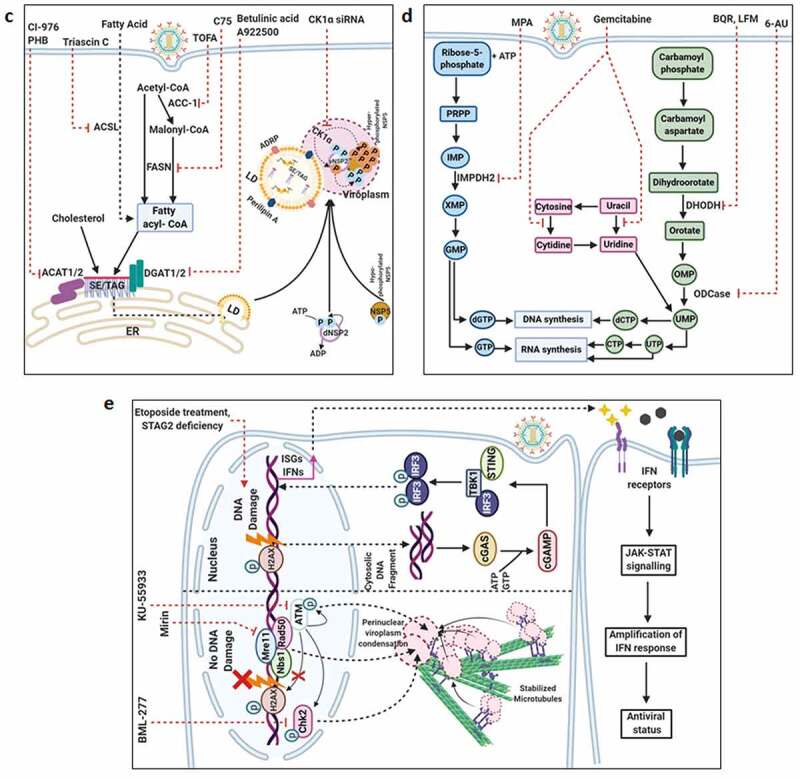
Continued

**Figure 4.**
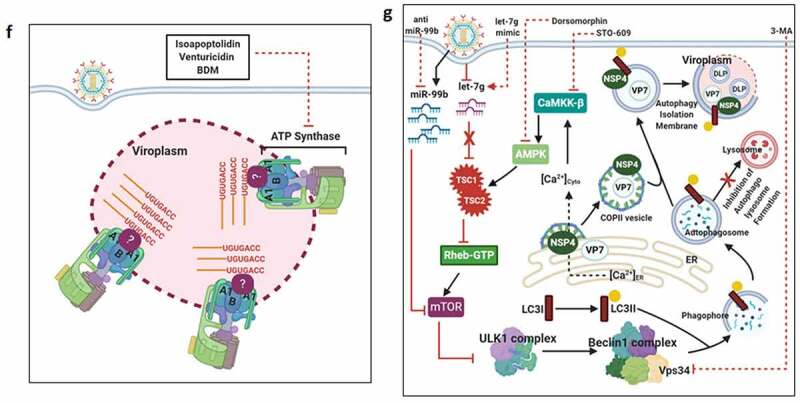
Continued

Another important host–RV interaction for regulation of biased synthesis of viral proteins at the expense of cellular ones include a high-affinity interaction between NSP3 with eIF4G, leading to displacement of a low affinity interacting partner PABPC1 from eIF4G [195–197]. Efficient translation initiation of eukaryotic mRNAs requires mRNA circularization through interaction between eIF4E (cap-binding protein at the 5ʹ termini) and PABPC1 [Poly(A) tail binding protein at the 3ʹ termini] via an intermediate scaffold eIF4G (Figure 4a). In absence of PABPC1 bound to poly(A) tail, circularization of cellular mRNAs through a eIF4E-eIF4G-PABPC1 interaction is abolished which might result in severe suppression of host cellular translation. Evicted PABPC1 is localized to nucleus within RV-infected cells, possibly facilitated via NSP3-Rotavirus X protein associated with NSP3 (RoXaN) interaction; PABPC1 nuclear retention in turn triggers hyperadenylation of cellular mRNAs thereby impairing nucleo-cytoplasmic mRNA export (Figure 4a) [193,198]. NSP3 mutants which are either incapable of binding to eIF4G or to RoXaN have reduced PABPC1 nuclear re-localizing property [198]. At the same time, NSP3, by interacting with the 3ʹ end of RV transcripts as well as with eIF4G, has been shown to enhance translation of viral messages, thereby functioning as a PABPC1 analogue (Figure 4a) [199]. Surprisingly, silencing NSP3 expression had no inhibitory effect (but rather a stimulatory impact) on viral protein translation/progeny yield [200], and NSP3 mutant RVs with reduced PABC1 nuclear targeting capability have been reported to exhibit similar growth characteristics when compared to wild type counterparts [201], suggesting dispensability of NSP3 for viral protein translation. Reports of NSP3 to act as translation surrogate for PABPC1 during cellular protein translation have also questioned the host translation inhibitory impact of NSP3 [199].

Production of huge number of viral transcripts (in the range of 10s of 1000s of molecules per cell, which is only about 10 times short of the amount of 18S rRNA present in the cell) coupled to the stagnation of cellular mRNA translation within RV-infected cells ensure overhauling of the host cellular translation machineries by the viral messages, which in turn amplifies host translation inhibition effects [193,202].

### Sequestration of RNA binding proteins at non-canonical niches: A “sponging” effect

Interestingly, recent reports have shown nucleus-to-cytosolic re-localization of many heterogeneous nuclear ribonucleoproteins (hnRNPs) and AU-rich element-binding proteins (ARE-BPs) along with their sequestration to viroplasms within RV-infected cells (Figure 3g). Interestingly, these translocation events were not observed in viroplasmic protein transfected cells, suggesting involvement of multiple viral components to account for changes in sub-cellular proteome during progressive RV infection. Moreover, many of the re-located hnRNPs (hnRNPs C1/C2, E, F/H, I, K/J, L, and U) and ARE-BPs (hnRNP D, BRF1, HuR, TIA1, TIAL-1, TTP, Staufen1, and KSRP) were found to get engaged in RNA-independent interactions with viroplasmic proteins NSP2 and NSP5 (Figure 3g) [192]. In depth molecular mechanisms for these translocation events have not been addressed yet. Surely, the possibility of a stochastic effect in the form of RV-induced loss of nuclear membrane integrity has been nullified. Interestingly, changes in the sub-cellular levels of nucleo-cytoplasmic transport factors have also been observed in RV-infected cells as infection triggered time point-dependent increase of Exportin1, Importin-β, Ran in cytosolic fractions and reduction of Transportin1 in nuclear fractions [192]. Involvement of these nucleo-cytoplasmic transport factors in regulating sub-cellular partitioning of proteins in RV-infected cells, however, awaits experimental affirmation. Based on the observations that some of these RBPs interact with viral RNAs and that nucleus-to-cytosolic shuttling of these proteins is sensitive to viral RNA depletion, a “sponging” effect of abundant viral RNAs to account for sub-cellular re-localization of host proteins has been put forward (Figure 3g) [156]. Indeed, RV mRNAs present 57 to 68% A + U content with UU, UA, and AU sequences being uniformly distributed along the mRNA length, suggesting possibility of absorbing re-located ARE-BPs [192]. Direct implications of such interaction on RV translational repression and/or destabilization are yet to be addressed. Nonetheless, significance from the viral perspective is evident as, RNAi-mediated silencing and plasmid-based overexpression of HuR, hnRNP D, hnRNP I, and hnRNP K led to diminished and increased progeny virus production, respectively. Other components (G3BP1, TIA1, and hnRNP C1) showed antiviral potency as their down-regulation facilitated RV infection and ectopic overexpression antagonized progeny virus yield [192]. Of note, nuclear exit of hnRNPs and ARE-BPs in context of RV infection also hints at an alteration in the nuclear RNA splicing and surveillance landscape within host cells, experimental validation of which would be of extreme interest in future.

### Regulation of Calcium homeostasis: A pro-rotaviral measure with diarrheagenic importance

As Ca^2+^ ions are important second messengers controlling a plethora of intracellular signaling cascades, Ca^2+^ homeostasis is often targeted by viruses as a part of virus-induced obligatory intracellular reprogramming [203,204]. Elevation of cytoplasmic calcium concentration ([Ca^2+^]_cyto_) plays an integral part in the pathophysiology of RV-induced diarrhea which is multifactorial in nature with both viral triggers and host mediators being essential for the complex disease outcome.

The diarrhea-causing viral component has been identified to be NSP4 both in its classical full length intracellular form (iNSP4) and shortened secretory extracellular form (eNSP4) [205–208]. iNSP4 regulates [Ca^2+^]_cyto_ elevation in RV-infected cells whereas eNSP4 has been implicated in [Ca^2+^]_cyto_ increase within uninfected bystander cells residing adjacent to RV-infected cell population [205,207,209]. Further corroborations revealed iNSP4 to have an intrinsic ion-channel activity and therefore to act as a viroporin [small, hydrophobic protein containing a cluster of basic residues (Lys or Arg) and an amphipathic-α-helix that oligomerizes to create a transmembrane aqueous pore] in the ER resulting in Ca^2+^ leakage from the ER store house and subsequent Ca^2+^ infusion through the plasma membrane (Figure 4b). The viroporin domain in iNSP4 has been narrowed down to the 47–90 amino acid region. Indeed, silencing NSP4 expression as a whole or mutation of either the cluster of basic residues or the amphipathic α-helix within NSP4 drastically abolished [Ca^2+^]_cyto_ elevation. Interestingly, the process operating behind NSP4-induced [Ca^2+^]_cyto_ increase has been shown to be biphasic-an initial Ca^2+^ loss from the ER through the constitutive viroporin activity of iNSP4 and a second phase of store-operated calcium entry (SOCE) involving the ER calcium sensor stromal interaction molecule 1 (STIM1) and a variety of calcium-release-activated calcium (CRAC) channels, including Orai1 and TRPC channels in the plasma membrane (Figure 4b). Low ER Ca^2+^ concentration has been reported to activate and oligomerize Ca^2+^-sensing ER transmembrane glyco/phosphoprotein STIM1 resulting in STIM1 oligomer translocation to the ER–plasma membrane junctions and subsequent stimulation of the CRAC channels (Orai1 and TRPC channels) for SOCE [210–212]. Knocking down STIM1 expression exerted significant antagonistic effects on RV progeny yield [211]. Using MA104 cell line stably transfected with dynamic fluorescent protein-based Ca^2+^ sensors, RV-induced dynamic [Ca^2+^]_cyto_ dysregulation has recently been identified at a single cell resolution over an extended period of time (from early-to-late time points of infection) [213]. Interestingly, instead of a monophasic sustained response which has been observed across a population of infected cells, this study strongly advocated for an oscillatory nature of the [Ca^2+^]_cyto_ regulation within a single cell which is manifested in the form of hundreds of discrete NSP4 viroporin-sensitive [Ca^2+^]_cyto_ spikes with a maximal response coinciding with the infection peak. Blockade of SOCE (by 2-APB, BTP2, Synta66, GSK7975A) mimicked anti-RV effects of STIM1 silencing by decreasing both the number of calcium spikes per cell and the amplitude of individual spike (Figure 4b) [213]. Apart from Orai1 and TRPC, potential contribution of the sodium/calcium exchanger NCX has been implicated in regulating Ca^2+^ homeostasis in RV-infected cells (Figure 4b). Under normal conditions, NCX forces Ca^2+^ out of cells using Na^+^ infusion down the gradient as the driving force. However, elevated intracellular Na^+^ levels (as seen in RV-infected cells) set in NCX to operate in reverse mode by pumping Na^+^ out and bringing Ca^2+^ into the cytoplasm. The inhibitory effect of KB-R7943, a blocker of NCX acting in reverse mode, supports the hypothesis of Ca^2+^ entry through NCX in its reverse mode in RV-infected or NSP4-expressing cells (Figure 4b) [214]. Unlike SOCE-blocker 2-APB, however, KB-R7943 only showed a modest decrease in the number of calcium spikes per cell but not in the spike amplitude [213]. There are conflicting reports on the involvement of voltage-gated calcium channels in RV-mediated Ca^2+^ dysregulation. As opposed to a previous study where partial sensitivity of RV-mediated [Ca^2+^]_cyto_ elevation to L-type voltage-gated channel blockade [by methoxyverapamil (D600)] was reported [215], no difference in the Ca^2+^ signaling either at the frequency or at the amplitude level was observed under D600 treatment regime when studied at single cell resolution [213]. Adopting different experimental protocols with differences in the cell lines and viral strains used, time points of observation chosen, and in the nature of Ca^2+^-sensing assays might explain this discrepancy. Nonetheless, crucial importance of SOCE as the main contributor of [Ca^2+^]_cyto_ elevation in RV infection scenario was also substantiated in monolayers of jejunam-derived human intestinal enteroid model. The degree of sensitivity to 2-APB, however, was less in enteroid system than in MA104 cell line, warranting further studies on RV-mediated Ca^2+^ dysregulation in the settings of heterogeneous cell population with a diverse Ca^2+^ channel repertoire [213].

Extracellular NSP4 (eNSP4), a secretory form of NSP4 released from the RV-infected cells in the extracellular milieu, has been implicated in elevating [Ca^2+^]_cyto_ in adjacent uninfected enterocytes and also in crypt cells (under *in vivo* condition) in an integrin-Phospholipase C (PLC)-Inositol 1,4,5-trisphophate (IP3)-dependent pathway (Figure 4b) [205,207,209,216]. To date, several forms of secretory NSP4 have been identified depending on the cell types, RV strains and methods of isolation. They include i) a 7 kDa non-classical Golgi-independent secretory product [208], ii) a glycosylated (Golgi-dependent secretion) detergent-sensitive oligomeric product assembled in a complex with phospholipids [217], and iii) a full-length, glycosylated, endoglycosidase H-sensitive form of NSP4 [218]. Secretory NSP4-dependent [Ca^2+^]_cyto_ elevation and subsequent activation of calcium-activated chloride channels (CaCC) (such as TMEM16A) leading to augmented Ca^2+^-dependent Cl^−^ secretion in the intestinal lumen has been considered to be a pivotal regulatory component of RV-induced secretory diarrhea [219] (Figure 4b). Consistently, inhibition of CaCC by plant products (Plumbagin from *Plumbago zeylanica* L., a herbal extract Krisanaklan, Shikonin from roots of *Lithospermum erythrorhizori*, resveratrol dimer trans-ε-viniferin and tetramer r-2-viniferin), red wine extracts, or targeted synthetic small molecule (CaCC_inh_-A01) proved to be significantly effective in attenuating intestinal fluid secretion without affecting rotaviral infection (Figure 4b) [220–224].

The host regulation of rotaviral pathophysiology includes a complex cross-talk between the absorptive enterocytes, the primary sites of RV infection, as well as the non-infected enterocytes, enterochromaffin cells (EC), crypt cells and the enteric nervous system (ENS), all of which become further interconnected by secretory products of viral (such as eNSP4) and host [neuroactive/hormonal substances such as 5-Hydroxytryptamine (5-HT) and vasoactive intestinal peptide (VIP)] origin [225–228]. Release of 5-HT, which modulates ENS-dependent secretory diarrheagenic response, vomiting reflexes, and intestinal motility, from EC cells has also been shown to be NSP4 (both iNSP4 and eNSP4)/Ca^2+^-regulated [225–228]. Mechanistically, granular discharge of 5-HT has been shown to stimulate ENS leading to release of VIP from nerve endings adjacent to crypt cells. VIP can elicit signaling cascade in crypt cells resulting in increased water and Cl^−^ secretion (Figure 4b) [226,227]. Indeed, the VIP receptor antagonist [(4Cl-D-Phe^6^, Leu^17^)-VIP] attenuated RV-induced diarrhea in mice model [229]. Use of anti-emetics such as Ondansetron and Granisetron (5-HT_3_ receptor antagonists) has also been clinically successful in alleviating acute gastroenteritis (Figure 4b) [230,231].

Apart from the direct diarrheagenic role, RV-induced [Ca^2+^]_cyto_ elevation has been implicated in intrinsic apoptosis induction within host cells [173], viroplasm formation [232], stabilization of VP7 trimers on the outer layer of progeny TLPs [7,233,234], re-organization of host cellular cytoskeletal networks [84] and initiation of a number of cellular signaling cascades of pro-rotaviral importance [27,235]. Core to mobilization of Ca^2+^-dependent signal transduction within RV-infected host cells has been a constitutively expressed 12 kDa Ca^2+^ binding protein Calmodulin (CaM) which at its Ca^2+^-bound state undergoes conformational shift to enable recruitment and activation of CaM binding proteins and subsequent downstream signaling. Reports of such transduction cascades include i) activation of the Ca^2+^/CaMKK-β/AMPK-dependent autophagic signaling (where autophagic membranes redirect ER-derived NSP4/VP7-containing vesicles to maturing DLPs within viroplasms) [27], and ii) induction of the Ca^2+^/CaMKI/Cyclin-Cdk/Rb/E2F signaling axis to facilitate G1-to-S phase transition of host cells [235] (required for RV replication) (Discussed in the following sections). Interestingly, increased protein level of CaM has been associated with RV infection (upto 8 hpi) and CaM was also found to interact directly with RV protein VP6 in a Ca^2+^-dependent way (Figure 4b) [236]. Not surprisingly, Ca^2+^ chelation (by BAPTA-AM) and/or CaM inhibition (by W7) proved to be antagonistic for RV propagation [236], suggesting immense pro-rotaviral significance of [Ca^2+^]_cyto_ elevation and Ca^2+^/CaM-dependent host cellular signaling (Figure 4b). With the recent discovery of Ca^2+^ dysregulation at the single cell level, correlation studies of Ca^2+^-dependent signaling events at the individual cell resolution can pave way for new dimensions to host-RV interactions and anti-RV therapeutics in future.

### Tuning the host cell cycle machinery with the viral rhythm

Cyclic progression of dividing cells through divisive (G1, S, G2, and M) and non-divisive stages (G0) is a strictly regulated phenomenon with presence of finely orchestrated intermittent checkpoints (G1-S, G2-M, intra-M). Besides their obvious implications in preventing tumorigenesis, cellular check point regulators are often targeted by viruses [237]. An intersection between RV infection and host cell cycle machinery has been reported from two independent studies. Both the studies concluded arrest of the cell cycle at S phase to produce an environment conducive to rotaviral replication independent of the RV strains and RV-permissive cell lines [235,238]. Prolonged intra-S phase retention observed in the presence of actively replicating RV is enabled by i) heralding the G1-S phase transition [235] and also by ii) impending entry into the M phase (Figure 4b) [238]. Pro-viral implications of prolonged S phase duration can be multifaceted such as stabilization of microtubular network for maintenance and maturation of viroplasmic structures, increase in host replication proteins which can potentially be usurped during viral replication, and generation of anti-apoptotic environment to ensure completion of viral replication cycle [235,238].

Augmented G1 to S phase transition in RV-infected cells was found to be accomplished by Retinoblastoma (Rb) hyperphosphorylation through the activities of specific Cyclin-Cyclin dependent kinase (Cdk) complexes [D type Cyclins (Cyclin D1, Cyclin D3) bound to CDK4 and CDK6; Cyclin E1 complexed with CDK2] and subsequent release of E2F which further translocate to nucleus for trans-activating E2F-responsive genes required for S phase entry (Figure 4b). Notably, transcript and protein level elevation as well as enhanced activities of all these Cyclin-Cdks concurred with RV-induced G1 to S phase transition in infected host cells. Interestingly, activation of Calcium/Calmodulin dependent protein kinase I (CaMKI) in response to RV-induced elevated Ca^2+^ concentration and CaM upregulation was subsequently identified to be the regulatory event upstream of Cyclin-Cdk-mediated Rb hyperphosphorylation (Figure 4b). Indeed, Ca^2+^ chelation (by BAPTA-AM) and CaM inhibition (by W7) efficiently reversed the CaMKI/Cyclin-Cdk/Rb/E2F signaling axis in RV-infected cells (Figure 4b). Pro-viral importance of S phase stasis was evidenced when viral protein expression and progeny yield was found to be higher in S phase synchronized cells (by AZT treatment) compared to unsynchronized control. Similarly, G0/G1 synchronized cells (by terfenadine treatment) showed reduced rotaviral infectivity when compared to either unsynchronized control and S phase synchronized group [235].

A recent report enunciated RV infection to arrest host cell cycle in S/G2 phase, thereby preventing transition on to the mitotic phase and prolonging S phase duration. Mechanistic studies revealed RV-induced down-regulation of Cyclin B1 expression and resulting unavailability of Cdk1-Cyclin B complexes to account for the blockade of mitotic entry. The viral regulators behind S/G2 arrest were identified to be three RV proteins NSP3, NSP5, and VP2 (Figure 4b) [238]. From the viral perspective, significance of M phase blockade has been hypothesized to be the prevention of microtubule disassembly (which generally coincides with mitotic entry) and usurping stabilized microtubular network for viroplasm formation/maturation and productive viral replication (Figure 4b) [238,239]. Indeed, the dynamicity of viroplasmic puncta, as evidenced by merging of these inclusion bodies with each other to form bigger and fewer aggregates at perinuclear locations, concurred with increased tubulin acetylation within infected cells [239]. Agreeably, interfering with the host cellular microtubular network (by nocodazole) and with the microtubular motor protein kinesin Eg5 (by monastrol) perturbed viroplasm dynamics (with respect to both spatial positioning within infected host cells and temporal condensation process) and caused reversal of RV-induced S/G2 cell cycle arrest (Figure 4b) [238,239].

### Casein kinase and rotaviral viroplasms: An orchestrated cascade of phosphorylation events

Besides host regulations for maintaining viroplasmic architecture and dynamicity, the formation of viroplasms also requires host cellular Casein kinase-mediated phosphorylation events of the viroplasm-nucleating proteins NSP2 and NSP5 (Figure 4c) [17,19,20,240–244]. Indeed, co-localization of Casein Kinase 1α (CK1α) with NSP2 and NSP5 within nucleating viroplasms has been observed [241,244]. Consistently, silencing the expression of CK1α drastically affected viroplasm formation and RV progeny production (Figure 4c) [244]. Detailed mechanistic profiling suggested that viroplasm nucleation event initiates with the auto-phosphorylation of the cytoplasmically dispersed NSP2 (dNSP2) population followed by its association with the nascent hypo-phosphorylated NSP5 (26 kDa). Subsequently, a series of CK1α-regulated phosphorylation reactions on both NSP2 and NSP5 result in gradual NSP5 hyperphosphorylations (isoforms at 28 kDa and above representing hyper-phosphorylated NSP5), association with lipid droplets, and higher order assembly structure formation (Figure 4c) [243,244]. Implications of CK-II-mediated phosphorylation events have also been suggested for NSP5 to form higher order oligomeric complex [245].

### Lipid droplets and rotaviral viroplasms

Lipid droplets (LDs) which are the principal cellular storage sites for sterol esters and triacylglycerols within a monolayer of phospholipid with characteristic protein inserts such as adipose differentiation-related protein (ADRP) and perilipin A, may also shape viral pathogenesis and therefore are at an important cross-section between the host cellular lipid metabolism and viral infection [246,247]. A strong positive correlation between rotaviral replication and host cellular lipid homeostasis has been reported especially with respect to contribution of LDs on viroplasm formation (Figure 4c) [248]. More precisely, certain conformational changes in NSP2 or NSP5 during viroplasm nucleation may expose lipophilic residues (NSP5 possesses an amphipathic helix) of the proteins which might further be inserted into the LD membranes [244]. In fact, taking the advantage of delayed viroplasm dynamicity in cells infected with recombinant RV with a phosphomimetic NSP2, a very recent report highlighted the association of nascent LDs with phosphorylated viroplasmic NSP2 even in the absence of NSP5 (especially the hyperphosphorylated form of NSP5) [249].

Several lines of evidences suggest a pro-rotaviral significance of cellular LDs in regulating viroplasms within infected cells-i) co-localization of NSP2 and NSP5 with perilipin A and ADRP within infected cells (Figure 4c) and also in NSP2-NSP5 co-expressing cells [248], ii) sensitivity of LD recruitment (as measured by perilipin A localization) to viroplasm inhibition in RV-infected cells, iii) co-sedimentation of viral dsRNAs with NSP5 and perilipin A in the same low-density fraction when RV-infected cell extracts (detergent-free) were subjected to equilibrium ultracentrifugation through iodixanol gradients [248], iv) high sensitivity of viroplasm formation and viral progeny production to chemical perturbation of cellular LDs (Figure 4c). To date, several enzymatic targets belonging to the neutral lipid biosynthetic pathway [such as long chain acyl-CoA synthetase (ACSL), acetyl-CoA carboxylase 1 (ACC-1), fatty acid synthase (FASN) complex, diacylglycerol acyltransferases (DGAT1, DGAT2), acyl-coenzyme A (CoA):cholesterol acyltransferases (ACAT1 and ACAT2)] have been identified which can be inhibited by small molecules [ACSL by triacsin C, ACC-1 by 5-(tetradecyloxy)-2-furoic acid (TOFA), FASN by C75, DGAT by A922500 or betulinic acid, and ACAT by CI-976 or PHB] to diminish RV infectivity (Figure 4c) [248,250,251]. Of note, TOFA interfered with RV outer capsid assembly and showed drug synergism with C75 [250,252]. On a consistent note, augmenting LD fragmentation (by a combination of isoproterenol + isobutylmethylxanthine) antagonized RV replication and RV-induced cytopathy [248]. Corroborative mass spectrometry-based differential lipidome studies on RV-infected cell lysates have also revealed increased lipid content of different classes in the iodixanol gradients. Interestingly, the low-density fraction where peak of RV dsRNA genome resided along with the lipid droplet- and viroplasm-associated proteins was found to contain elevated amounts of lipids which are concentrated in lipid droplets, confirming lipid droplets to associate with viroplasms in RV infected cells [253]. Moreover, activation of farnesoid X receptor (FXR) by natural ligands bile acids [such as chenodeoxycholic acid (CDCA)] or by synthetic agonists (such as GW4064) led to reduction of cellular triglyceride contents and concomitant attenuation of RV replication. Oral administration of CDCA in mice significantly restricted fecal virus shedding [254].

### Interfering with the host cellular nucleotide metabolism

Utilization of host nucleotide resources (purines and pyrimidines) is an absolute requirement for viral transcription and replication. Not surprisingly, therefore, *de novo* biosynthesis and salvage pathways of nucleotide generation have emerged as sensitive antiviral targets [255,256]. Indispensability of host cellular nucleotide pool for RV is also evidenced when rotaviral replication became heavily sensitized both in cell lines as well as in human intestinal organoid model upon pharmacological inhibition of enzymes involved in the nucleotide biosynthesis pathway. Examples in this regard include anti-RV effects observed through inhibition of dihydroorotate dehydrogenase (DHODH) [by brequinar (BQR) and leflunomide (LFM)], and orotidine 5′-monophosphate decarboxylase (ODCase) [by 6-azauracil (6-AU)] in the *de novo* pyrimidine biosynthesis [257], and of inosine monophosphate dehydrogenase 2 (IMPDH2) [by mycophenolic acid (MPA)] in the *de novo* purine biosynthetic pathway (Figure 4d) [258]. Moreover, inhibition of salvage pathway of pyrimidine biosynthesis (by gemcitabine) also proved derogatory for RV propagation (Figure 4d) [259]. Interestingly, anti-RV impacts of all these inhibitors were abolished when depleted nucleotide pool was replenished exogenously [257–259]. Therefore, interfering with the host nucleotide metabolism by depleting host nucleotide pool can be an effective anti-RV measure.

### Pro-rotaviral implication of Rac1 GTPase

Rac1 belongs to the Rho family of GTPases and is involved in crucial cellular signaling pathways including actin microfilament redistribution and gene transcription [260]. The significance of biologically active Rac1 (Rac1 bound to GTP) in fostering RV replication was substantiated when treatment with Rac1 inhibitor (NSC23766, 6-Thioguanine), Rac1 silencing through RNAi, or ectopic overexpression of dominant negative Rac1 mutant effectively curtailed RV RNA yield [87,261]. This is of particular clinical relevance as 6-Thioguanine is used routinely as immunosuppressive agent in organ transplant recipients and inflammatory complications (auto-inflammatory bowel disease) where nosocomial RV infection is common [261]. Though the exact mechanism by which Rac1-GTP regulates RV RNA production is yet to be ascertained, implications of several Rac1–dependent signaling cascades such as p38MAPK/JNK and PI3K-Akt-mammalian target of Rapamycin (mTOR) have been given [87,262,263].

### Hijacking the ubiquitin-proteasome system

Proteolytic ubiquitylation and degradation by 26S proteasome regulate protein turn over. Two independent studies reported inhibition of proteasome either by chemical inhibitors or by RNAi to have antagonistic effects on rotaviral RNA and protein synthesis as well as on the yield of infectious viral progeny [264,265]. Moreover, this antagonism was found to be independent of viral entry, stochastic interferon signaling, and *in vitro* polymerase activity of VP1. The exact mechanism by which proteasome activity regulates RV replication is not well understood. In one study, proteasome inhibition was observed to be accompanied by compromised viroplasm formation [264]. Proteasome inhibition also led to failure of VP1, VP2, and VP6 to be effectively incorporated into poorly formed viroplasmic puncta, partially explaining inhibitory effect of proteasome inhibitor on genome replication and infectious virus yield [265]. Interestingly, relevance of proteolytic ubiquitylation on RV replication was also revealed when ubiquitin overexpression in proteasome inhibitor-treated cells partially rescued viral yield [265]. Corroborative studies showed RV infection to trigger proteasomal degradation of an array of host substrates such as IRF3 [129,130,266], IRF5, IRF7, IRF9 [128,131], β-TrCP [124], Pan3 [189], AGO2 [154], TRAF2 [120], MAVS [119], p53 [180], p62 [267], all of which are pivotal antiviral host determinants. There are reports available for proteasome-independent depletion of host proteins in RV-infected cells [116,134], ruling out possible stochastic effects of proteasome inhibitors during infection. RV-NSP1 is usually the viral trigger behind ubiquitin-proteasome-dependent demise of crucial host determinants. RV‐NSP1 possesses a highly conserved N‐terminal RING domain with a zinc‐finger motif (C‐X2‐CX8‐C‐X2‐C‐X3‐H‐X‐C‐X2‐C‐X5‐C consensus), which has been postulated to have putative E3 ubiquitin ligase activity. RV‐NSP1 has also recently been reported to interact with [121,123,268] and degrade β‐TrCP by co‐opting Cullin-RING E3 ubiquitin ligases (CRLs) [123]. Therefore, hijacking ubiquitin-proteasome system for degrading antiviral host proteins can be an efficient strategy utilized by RV to ensure productive replication and perpetuation.

### Exploiting the host SUMOylation machinery

Posttranslational modification by small ubiquitin-like modifiers (SUMO), a process called SUMOylation, regulates functional flexibility of target proteins mainly by modulating protein stability and protein-protein interactions [269,270]. Interestingly, RV has been reported to hijack cellular SUMOylation machinery for SUMO-modification of several viroplasmic proteins such as VP1, VP2, NSP2, VP6, and NSP5. Moreover, NSP5 SUMOylation mutant underwent a higher degree of phosphorylation than its wild type counterpart and failed to form viroplasm-like structures when co-expressed with VP2. Proviral implications of host SUMOylation machinery on RV replication were further substantiated from two important observations-i) overexpression of SUMO isoforms (SUMO1 and SUMO2) led to enhanced viral protein synthesis as well as increased rotaviral replication, and ii) silencing of E2 SUMOylation enzyme Ubc9 by RNAi resulted in a marked attenuation of rotaviral proteins and subsequent viral progeny yield [271].

### Non-canonical usurpation of DNA damage response pathway

DNA damage response (DDR) is a highly conserved signaling cascade in eukaryotic cells to maintain genomic integrity. Canonical DDR includes sequential occurrence of events: initial sensing of DNA lesions (by a group of proteins called sensors) followed by transduction of the damage signal (via another group of cellular proteins named transducers) to the effectors for carrying out necessary changes. Upon activation, DDR culminates in either cell cycle arrest allowing cells to activate damage repair pathways for restoring genomic integrity or triggers apoptosis in case of irreparable lesions in the DNA [272–274]. Interestingly, RV infection was found to activate the transducer kinase ataxia telangiectasia mutated (ATM) and its downstream effector checkpoint kinase 2 (Chk2) (Figure 4e), with the activation response most heightened at 6 hpi. RV-induced ATM-Chk2 activation was revealed to be dependent on the induction of the upstream sensor Mre11-Rad50-Nbs1 (MRN) complex (Figure 4e). However, evidences of nuclear DNA damage (double-stranded DNA breaks) which usually precedes induction of ATM-Chk2 pathway and of formation of damage-induced canonical nuclear foci of λ-H2AX (Ser139 phosphorylated histone variant H2AX), a signature response at the sites of DNA lesions, were not observed in RV-SA11 infected cells at 6 hpi (Figure 4e). Subsequent investigations showed induced levels of ATM-Chk2 as well as of MRN components to get relocated from nucleus to cytoplasm and to get sequestered into the viroplasmic puncta (Figure 4e). Interestingly, inhibition of ATM and Chk2 by targeted small molecules (ATM inhibitor KU55933, Chk2 inhibitor BML-277) significantly abrogated fusion and maturation of viroplasms with progression of infection leading to heavily restricted rotaviral propagation (Figure 4e). Notably, occurrence of nuclear DNA damage and damage-induced nuclear foci were observed at later hours of infection (12 hpi) when maximal activation response of ATM-Chk2 pathway has subsided, indicating a temporal distinction between the canonical DDR and the virally manipulated skewed response [275].

Interestingly, genomic DNA damage due to deficiency of Stromal antigen 2 (STAG2), a component of the nuclear Cohesin complex, has been recently reported to show potent anti-RV effects by triggering the cyclic GMP-AMP synthase-Stimulator of Interferon genes (cGAS-STING)-dependent cytosolic DNA sensing pathway which further elicited JAK-STAT-mediated antiviral IFN signaling (Figure 4e) [140]. Moreover, etoposide, a DNA damage inducer, recapitulated anti-RV effects of STAG2 deficiency through augmentation of DNA damage response (including induction of λ-H2AX) and subsequent activation of IFN signaling (Figure 4e) [140]. This observation again substantiates a distinct demarcation between the occurrence of canonical DDR, which might be antiviral in nature, and the virally skewed response of pivotal pro-rotaviral significance.

### An energetic association: Host contribution in viral genome packaging

Involvement of host determinants in viral genome packaging within the core shell of the assembling progenies has remained elusive. Only recently, narrowing down from a RNA–protein interaction detection (RaPID)-based RV 3ʹ untranslated region (3ʹ UTR)-bound proteome study, a high-affinity interaction of ATP5B, a core subunit of the mitochondrial ATP synthase, with RV (Group A) 3ʹ UTR consensus (5ʹ-UGUGACC-3ʹ) was evidenced within viroplasms of infected cells [276]. ATP5B depletion through RNAi or chemical inhibition of ATP synthase holoenzyme (by isoapoptolidin, venturicidin, BDM) heavily restricted RV progeny yield by sensitizing late stage of RV life cycle events such as viral genome assembly (Figure 4f). Anti-RV effects of isoapoptolidin at the stage of RV progeny production were also mimicked in intestinal organoid model of infection. Consistently, the proteomics screening also identified two other subunits of ATP synthase complex, ATP5A1, and ATP5O, as bonafide RV (Group A) 3ʹ UTR interactors (Figure 4f), and silencing these subunits reiterated anti-RV effects of ATP5B depletion. To address the functional significance of such cellular ATPase machinery recruitment from mitochondria in uninfected cells to 3ʹ UTR of RV RNAs upon infection, a hypothesis has been put forward where ATPase-driven critical energy might foster viral genome packaging. However, failure of ATP5B to shift the mobility of RV (Group A) 3ʹ UTR consensus in an electrophoretic mobility shift assay implies a possible indirect interaction through the involvement of intermediate candidates such as VP1 which accumulates during infection and has high affinity for the consensus [276].

### Carrying the viral burden: Utilizing the autophagic membranes to transport rotaviral protein cargo

Macroautophagy is a finely tuned catabolic process, which involves the formation of double-membrane-bound vesicles called autophagosomes containing engulfed cargos (damaged organelles, long-lived proteins, intracellular pathogens) and channeling the membrane-bound vesicles through an intracellular membrane trafficking pathway to culminate in lysosomal compartment for degradation of the engulfed contents. Because of its reported contribution on pathogen elimination, autophagy often becomes a sensitive target of subversion or exploitation by viruses [277,278]. Several observations from independent groups have documented a close interaction between RV infection and host cellular autophagy [27,267,279]. A unifying theme holds pro-viral implication of autophagy during RV infection as suppression of important autophagy genes (LC3, Atg3, Atg5, Atg7) or pharmacological inhibition of autophagosome formation [by using 3-methyladenine (3-MA) which targets Vps34, a type III PI3K belonging to the Beclin-1 complex and required for autophagosome formation] resulted in significant attenuation of viral progeny yield (Figure 4g) [27,267]. Presence of two triggers has been identified to date to regulate autophagy initiation. One of these stimuli requires NSP4-mediated increase of cytosolic Ca^2+^ concentration and subsequent activation of calcium/calmodulin-dependent kinase kinase β (CaMKK-β) and AMP activated protein kinase (AMPK) in a sequential manner for initiation of autophagy (Figure 4g). Quenching cytosolic Ca^2+^ (through Ca^2+^-chelator BAPTA-AM) and/or inhibiting CaMKK-β (by targeted small molecule STO-609) proved derogatory for autophagy initiation and productive rotaviral replication (Figure 4g) [27]. Significance of AMPK as a crucial pro-rotaviral determinant has further been evidenced when pharmacological inhibition of AMPK (by dorsomorphin) reduced the number of VP6-expressing cells and VP6-intensity (Figure 4g); consistently, a direct activation of AMPK (by using the AMP analog AICAR) increased the proportion of late-stage infected cells, suggesting AMPK activation to accelerate RV infection progression [190]. However, targets of activated AMPK have been hypothesized to extend beyond autophagy pathway to a whole range of cellular processes, including organelles rearrangement, lipid store consumption, stagnated host gene transcription/translation, and cell cycle transition [190].

Another miRNA-regulated mechanism, independent of the NSP4/Ca^2+^/CaMKK-β/AMPK pathway, has been reported very recently to contribute to autophagy initiation in RV-infected cells where synchronized modulation of miR-99b and let-7 g was found to promote Tuberous sclerosis protein 1/2 (TSC1/2)-mTOR-dependent autophagic signaling (Figure 4g) [279]. Briefly, RV infection was evidenced to downregulate miRNA let-7 g which in turn elevated the expression of let-7 g-target TSC1. Subsequent stabilization of TSC1-TSC2 complex suppressed levels of Ras homolog enriched in brain-GTP (Rheb-GTP) as TSC2 acts as a GTPase-activating protein (GAP) toward Rheb. Rheb-GTP itself is a positive regulator of mTOR. Therefore, in presence of stabilized TSC1-TSC2 complex in RV-infected cells, mTOR activity was restricted. A concomitant mTOR suppressive mechanism emerged when mTOR was revealed to be the direct target of the RV-induced miRNA miR-99b. Synchronized modulation of these two miRNAs (down-regulation of let-7 g and up-regulation of miR-99b) in RV-infected cells restricted mTOR expression and activity which culminated in induction of autophagy (Figure 4g). Importantly, overexpression of let7g (by let-7 g mimic) together with miR-99b inhibition (by anti-miR-99b) resulted in marked reduction of autophagy markers (such as LC3 II, Beclin1, and Atg5) leading to heavily restricted rotaviral RNA/protein expression and titer (Figure 4g) [279]. Moreover, treatment of RV-infected cells with STO-609 or miRNA cocktail (let-7 g mimic together with anti-miR-99b) alone has less impacts on autophagy inhibition and subsequent RV replication than when a combinatorial treatment regime was adopted, suggesting the two stimuli to possibly act in an additive yet mutually exclusive fashion during RV infection scenario [279].

Interestingly, RV-induced autophagic isolation membranes has been reported to be hijacked from being channeled to the canonical lysosomal degradation pathway to ultimately facilitate ER-to-viroplasm transportation of viral proteins NSP4 and VP7 for production of progeny TLPs (Figure 4g) [27,28]. Consistently, the presence of NSP4 and VP7 to reside surrounding autophagosome-engulfed viroplasms was proved to be heavily sensitive to autophagosome inhibition by STO-609 [27]. Recent follow-up studies reiterated RV-induced autophagy to have a crucial involvement in redirecting NSP4- and VP7-containing ER-derived Coat protein complex II (COPII) vesicles from Golgi-apparatus (which are the canonical destination of ER-derived COPII vesicles) to DLPs within viroplasms (Figure 4g) [28]. A summarized view of sequential events can be given as-i) exit of NSP4 (by virtue of its interaction with the COPII cargo binding protein Sec24) into COPII vesicles from ER (an autophagy protein independent event), followed by ii) hijacking of the COPII vesicles by LC3 II positive autophagic membranes (an autophagy-dependent event facilitated by NSP4-LC3 interaction) leading to stripping of the ER membrane markers (SERCA or calnexin), and iii) redirection of the NSP4/LC3 II-containing membranes to viroplasms (Figure 4g). Interfering with the COPII vesicle formation/release from ER (either by inhibiting Sar1, a small GTPase regulating initiation of COPII vesicle formation, through overexpression of its dominant-negative GDP-restricted form or by RNAi-mediated silencing of CK-II which phosphorylates Sec31, an outer coat protein around COPII vesicle) abrogated NSP4 translocation around viroplasms, leading to reduced production of infectious TLPs [28].

Intriguingly, vitamin D_3_ metabolite has been shown to inhibit porcine RV infectivity by augmenting autophagic signaling and modulating autophagolysosome formation as well as expression of cathelicidins [280]. Therefore, re-orienting NSP4 containing autophagic isolation membrane to lysosomal demise can also be an effective anti-RV measure as it might promote clearance of viral components. On a consistent note, another recent report contradicted the pro-rotaviral significance of autophagy where inhibition of mTOR (by rapamycin) or PI3K (by LY, 294–002) individually or in combination (by BEZ235) was found to trigger 4E-BP1-dependent induction of autophagy and concomitant reduction of rotaviral titer [263,281]. Differences in cell lines and rotaviral strains used and infection time points studied might explain this discrepancy; however, detailed follow-up studies for such justification are lacking.

### Host contribution in rotaviral morphogenesis and exit

Rotaviral morphogenesis requires a budding step through the cytoplasmic cellular membranes of ER origin for acquiring the glycoproteinaceous VP7 layer. Significance of ER chaperones in the morphogenesis of infectious RV particles was evidenced when knocking down the expression of GRP78, protein disulfide isomerase (PDI), calnexin, and calreticulin, (but not of GRP94 and ERp57) halfened the yield of infectious virions possibly by impairing the virion assembly step [282]. Though the exact mode of interaction was not addressed, an implication of the chaperones in promoting correct and sequential post-translation modifications of ER-glycosylated RV proteins VP7 and/or NSP4 was given; indeed, physical association of ER chaperones with NSP4/VP7/VP4 has been reported [283–285].

A recent report demonstrated how the host cellular guanine nucleotide exchange factor GBF1 (Golgi-specific brefeldin A-resistance guanine nucleotide exchange factor 1), canonically involved in the Coat protein I/ADP ribosylation factor 1 (COPI/Arf1)-mediated vesicular transport, can be usurped by RVs to promote outer capsid assembly as a part of the viral progeny morphogenesis [286]. Mechanistic profiling revealed that loss-of-function of GBF1 [through RNAi or by brefeldin A (BFA)/Golgicide A (GCA) treatment], but surprisingly not of Arf1, resulted in significant attenuation of RV progeny yield by impairing VP7 trimerization and assembly onto nascent DLPs. Moreover, an altered posttranslational modification pattern of NSP4 in response to GBF1 silencing/inactivation was implicated behind such failure of VP7 trimerization. Whether the GBF1/COPI-mediated vesicular transport (possibly involving GBF1 substrates other than Arf1 such as Arf4/5) at the ER-Golgi interface has direct involvement in regulating RV outer capsid assembly within ER lumen or has some secondary implications in promoting non-canonical transport between ER and LDs or involving ER Golgi intermediate compartment (ERGIC) has remained unanswered [286,287]. Indeed, participation of ERGIC in late stages of TLP assembly has been implicated when VP4 and virion-assembled VP7 were found to co-localize with ERGIC resident protein ERGIC-53 [36]. In fact, physical association of VP4 with lipid raft microdomains has suggested a possibility of post-ER occurrence of VP4 assembly onto VP7-containing virion particles either within the raft microdomains or in ERGIC compartments [36,37]. On a consistent note, perturbing lipid raft integrity through membrane cholesterol depletion (by Methyl-β-cyclodextrin) or inhibition of cholesterol biosynthesis (by HMG-CoA reductase inhibitor lovastatin) significantly affected TLP assembly and non-lytic virion release [59,288,289]. Emerging evidence suggests non-lytic release of virions to occur also in non-polarized cells [290], indicating importance of lipid raft integrity and lipid raft-targeting of VP4 in these cell lines. Furthermore, non-lytic RV egress from non-polarized MA104 cells and final RV morphogenetic steps were revealed to be sensitive to actin inhibitor jasplakinolide, suggesting involvement of an actin-dependent mechanism [290].

Another study has also enunciated non-lytic release of vesicle-cloaked rotaviral particles within extracellular vesicles and integrity of such vesicles to remain intact during the procedure of fecal-oral transmission [291]. This viral clustering has been shown to effectively increase RV multiplicity of infection for successive waves of infection [291]. A very recent report substantiated this finding where RV infection was shown to be accompanied with increased vesicle secretion and non-lytic release of viral particles associated with these microvesicles both externally and internally [292]. However, whether RVs actively hijack cellular secretory pathway in favor of apical viral release or merely access a preexisting secretory stream for dissemination is yet to be ascertained.

## Antiviral HOSTility: A future of antiviral therapeutics to ponder upon

The expanding knowledge on multifaceted host-RV interactions has clearly elucidated how host repertoire is used or abused during the course of infection to enable progression of the viral life cycle within host cells. Sketching a global landscape of such interactive networks also has an added advantage as it provides a blueprint where from important host determinants can be identified and further be targeted therapeutically. Notably, in all the reports where host-targeted small molecules have been used to curtail rotaviral infectivity, the possibility of stochastic bystander cytotoxicity has been nullified. Importantly, a few of such host components (such as PI3K, AMPK, Ca^2+^/CaM) have been shown to be involved in multiple stages of RV life cycle and therefore might serve as ideal druggable candidates. On a similar note, many host factors and signaling pathways (PI3K/AKT/mTOR, AMPK, Ca^2+^/CaM, LD biogenesis, endosomal acidification, MRN/ATM/Chk2, ubiquitin-proteasome and SUMOylation system, purine/pyrimidine biosynthesis, Nrf2/ARE signaling, regulators of IFN signaling) are involved in regulating the infection cycles of viruses other than RV; targeting of such determinants, therefore, has broad antiviral (and sometimes even antimicrobial) relevance. Again, narrowing down on some host targets whose therapeutic intervention has been clinically proven in some other disease dimension has the most promising therapeutic potential. Indeed, such drug repurposing offers sumptuous risk-versus-reward trade-off with costs, risks and timeline of drug identification substantially reduced compared to the conventional drug discovery [293,294]. Existing anti-diarrheal drug therapy is mostly host-directed: use of anti-emetics (such as metoclopramide, dimenhydrinate and ondansetron) which decrease the number of vomiting episodes and the requirement of intravenous rehydration [230,231], and of anti-secretory racecadotril (an intestinal enkephalinase inhibitor) that reduces the secretion of water and electrolytes into the gut [295–298]. Virus-directed curative dimensions have also been tested. Notable examples are antiviral drug therapy with nitazoxanide which reduced duration of diarrheal episodes in children suffering from acute rotaviral diarrhea possibly by interfering with the viral morphogenesis [299–302] and with smectite (diosmectite; a natural alumino-silicate clay) that adsorbs infectious viral particles [303–305]. Moreover, host factor-independent targeting of RV has also been reported *in vitro* using other small molecules with viroplasm/DLP disintegrating potency [306] and viral transcription inhibitory effects [307]. With the recent advances in technological refinement such as adopting human intestinal organoids as infection model [86,123,140,141,213,257–259,261,263,276,308,309] and using rotaviral reverse genetics [187,245,249,310–313], future research should be propelled toward unraveling novel mechanistic aspects of host–RV interactions, and assessing therapeutic potential of reported anti-RV small molecules (host-targeted, virus-targeted, or in potential synergistic combination) in clinical settings.
